# Dynamic intercellular transport modulates the spatial patterning of differentiation during early neural commitment

**DOI:** 10.1038/s41467-018-06693-1

**Published:** 2018-10-05

**Authors:** Chad M. Glen, Todd C. McDevitt, Melissa L. Kemp

**Affiliations:** 1 The Wallace H. Coulter Department of Biomedical Engineering, Georgia Institute of Technology and Emory University, Atlanta, GA 30332 USA; 2 Gladstone Institute of Cardiovascular Disease, San Francisco, CA 94158 USA; 3 Department of Bioengineering & Therapeutic Sciences, University of California, San Francisco, CA 94158 USA

## Abstract

The initiation of heterogeneity within a population of phenotypically identical progenitors is a critical event for the onset of morphogenesis and differentiation patterning. Gap junction communication within multicellular systems produces complex networks of intercellular connectivity that result in heterogeneous distributions of intracellular signaling molecules. In this study, we investigate emergent systems-level behavior of the intercellular network within embryonic stem cell (ESC) populations and corresponding spatial organization during early neural differentiation. An agent-based model incorporates experimentally-determined parameters to yield complex transport networks for delivery of pro-differentiation cues between neighboring cells, reproducing the morphogenic trajectories during retinoic acid–accelerated mouse ESC differentiation. Furthermore, the model correctly predicts the delayed differentiation and preserved spatial features of the morphogenic trajectory that occurs in response to intercellular perturbation. These findings suggest an integral role of gap junction communication in the temporal coordination of emergent patterning during early differentiation and neural commitment of pluripotent stem cells.

## Abstract (web summary)

How heterogeneities arise in stem cell populations remains unclear. Here, Glen et al. find that in ESC colonies cell cycle asynchronies modulate gap junctions, causing variation in intracellular signalling molecule diffusion between cells, and ultimately in spatial heterogeneity in differentiation.

## Introduction

The spatial organization of heterogeneous cells within multicellular systems, such as tissues and organs, is a primary determinant in deriving their respective functionality^1^. During embryogenesis, pluripotent cells migrate and differentiate to form complex multicellular structures in a reliable and reproducible manner. An incomplete understanding of the dynamic signaling mechanisms that affect differentiation and morphogenic patterning limits faithful and accurate replication of emergent behavior in vitro.

To create more sophisticated engineered living systems (ELS), it is necessary to elucidate the collective impact of the numerous processes that shape multicellular constructs during normal development. Embryonic stem cells (ESCs) are an excellent model system for mimicking aspects of embryonic morphogenesis and investigating the various modes of communication amongst pluripotent populations^2^. The process of secretion, diffusion, and uptake of molecules is a well-established mechanism of biochemical communication across tissues, with the formation of extracellular morphogen gradients providing positional information that instructs cell fate decisions during differentiation, both in vitro and in vivo^3–5^. However, emerging evidence in recent years suggests that direct cell-cell communication plays an equally significant role in pattern formation during morphogenesis^6–10^. Ascertaining the role of intercellular communication as a regulator of differentiation is crucial for deciphering the diversity of spatial cues present during developmental processes and for the future derivation of more complex ELS.

Gap junction communication (GJC) provides direct channels that facilitate intercellular diffusion of small molecules (<1 kDa) between the cytosol of adjacent cells. Gap junctions assemble from hemichannels of connexin proteins present in the plasma membrane of adjacent cells and the connexin composition of each channel dictates the permeability of specific metabolites^11^. Furthermore, the transcription and translation of connexin isotypes is regulated by cellular phenotype, allowing cells to exercise considerable dynamic control over intercellular connectivity during differentiation and tissue development^12^. The collective GJC across a population of cells produces an intercellular network of cells with fluid connectivity. The versatility of GJ-connectivity creates vast potential for the development of intracellular gradients of small molecules - such as cAMP, ATP, and serotonin - that influence many downstream metabolic and transcriptional processes governing cell-fate decisions^13–17^. Unfortunately, accurately interpreting molecular gradients within a network of differentiating ESCs is challenging due to the close-packed density of epithelial cells and development of gradients across various length scales. While some sensors are capable of discerning concentration gradients of small molecules, many rely on FRET-based detections and have noted limitations^18^. Specifically, bleed through of the FRET-donor can skew measurements and an inherently low signal-to-noise ratio severely limits the sensitivity of these sensors. Furthermore, while several techniques exist for characterizing GJ transport^19,20^, they typically offer limited capability to quantify fluctuations in connectivity at a single-cell resolution simultaneously with the transport behavior at the population level. The difficulty of quantifying the influence of individual cells on the intercellular network is compounded when considering connectivity that can both modulate and be modulated by dynamical differentiation processes occurring throughout the population. For such instances, computational modeling offers an attractive approach, in combination with single-cell transport data, to investigate the dynamics of multicellular GJ communication and its relationship with differentiation.

In this work, we quantified intercellular transport rates from single cells within ESC colonies, identified cell cycle state as a modulator of these rates, and used this knowledge to construct a computational model of intercellular transport in a multicellular system. This agent-based model, regulated by cell cycle and considering growth, division, and differentiation, generated a complex, dynamic network topology of communication that was capable of predicting spatiotemporal perturbations of Oct4 expression during early neural commitment. We quantified spatial patterns through dimension reduction techniques using derived network metrics to directly and quantitatively compare experimental results and simulation data; this approach enabled the development of accurate and predictive computational models for investigating communication within multicellular systems. Our findings highlight the importance of asynchronous cell division in establishing molecular gradients across tissue-scale systems. We provide a framework for investigating the spatial evolution of differentiation within multicellular systems and report the previously unrecognized capability of intercellular communication to delay differentiation.

## Results

### Spatial differentiation during retinoic acid treatment

To evaluate spatial patterning during neural differentiation, monolayers of murine ESC colonies were treated for 72 h with retinoic acid (RA, 1 μM), a potent morphogenic promoter of neural progenitor cells that emerge during hindbrain development^21^. A 2D monolayer system was selected to enable a more comprehensive link between the patterning produced and intercellular communication between neighboring cells. In particular, ESCs are known to secrete paracrine factors that can accumulate within the confines of an aggregate and affect differentiation in a spatial manner^22^. The 2D system minimizes this potential for environmental heterogeneity and consequently allows heterogeneity caused by intercellular mechanisms to be studied in a less confounded context. The progression of differentiation was assessed based on expression of the two pluripotency factors, Oct4 and Sox2. The Oct4 + Sox2 + state has been well characterized to reflect the pluripotent state of ESCs, whereas Sox2 expression alone (in the absence of Oct4) is associated with a neural ectoderm fate, indicating that the Oct4-Sox2 + phenotype is the initial neural progenitor state after loss of pluripotency^23–25^.

**Fig. 1:**
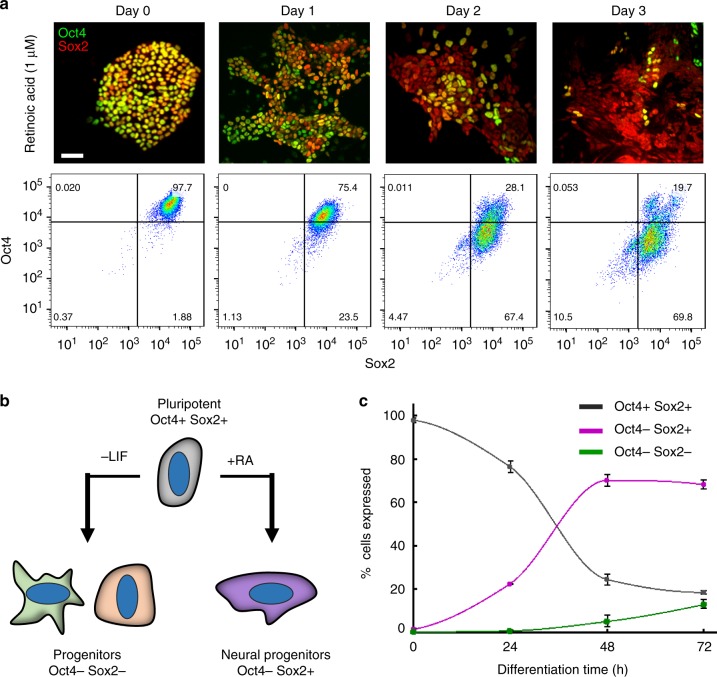
Loss of Oct4 and maintenance of Sox2 expression during retinoic acid–induced differentiation. Oct4 expression begins to decline after 24-hour exposure to retinoic acid (1 μM), with the main transition to an Oct4- state occurring between 24 and 48 h of RA treatment. After 72 h, ~ 70% of the population retain an Oct4-Sox2 + phenotype (**a**, **c**). A schematic depicting the change in gene expression in ESC populations towards the predominant progenitor state during two differentiation protocols: LIF-withdrawal in serum-containing media and retinoic acid addition (**b**). Quantification of flow cytometry data in **a** demonstrates the maintenance of the Oct4-Sox2 + state between 48 and 72 h of RA treatment (**c**), *n* = 9 (three biological replicates, three technical replicates for each biological replicate). Scale bar: 50 µm

As differentiation proceeded, a transient, patterned loss of Oct4 was observed while Sox2 expression was maintained, as expected for neural progenitors (Fig. 1a). The majority of the cell population (70 ± 4%) assumed an Oct4-Sox2 + neural progenitor state within 48 h of RA treatment. In contrast, spontaneous differentiation induced by LIF-withdrawal from serum-containing media resulted in a decrease in both Oct4 and Sox2 expression, associated with primitive endoderm and mesoderm specification (Fig. 1b, c and Supplementary Figure 1)^26^. RA-accelerated differentiation was used hereafter in this study due to the selective commitment of ESCs to the neural lineage. The selectivity provides a robust system for studying the emergence of spatial organization during the transition of a cell population from a pluripotent to a largely homogenous neural progenitor state.

### Differentiation causes a transient redistribution of Cx43

**Fig. 2:**
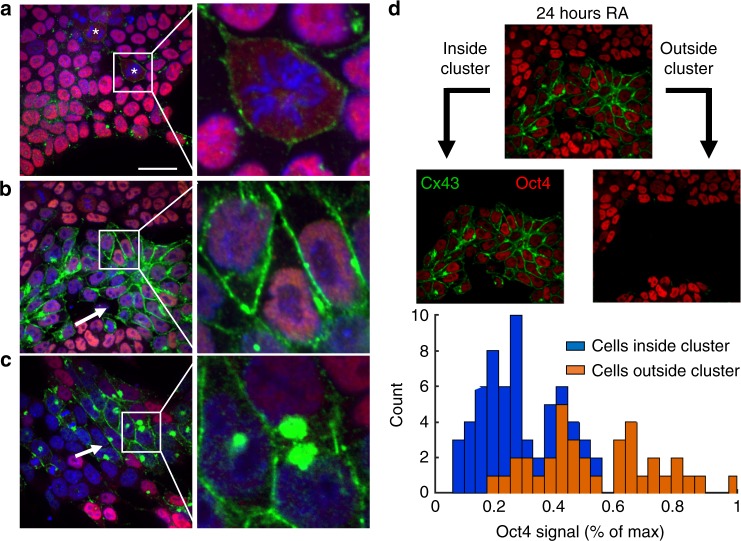
Cx43 signal (green) increases during retinoic acid–induced differentiation, with compartmentalization of transitioning cells between 24 and 48 h. Mitotic cells are prevalent in pluripotent colonies (**a**) and show a diffuse ‘ring’ of Cx43 in the membrane, designated by an asterisk, that is typical of Cx43 not forming GJ plaques. After 24 h of RA treatment (**b**), Cx43 noticeably increased between clusters of cells with low Oct4 expression (red), characterized in **d**. At 48 h of treatment (**c**), cells that have low (but non-zero) Oct4 expression have large expression of Cx43 in the cytoplasm. Previous studies have linked an accumulation of Cx43 expression in the cytoplasm to localization in the Golgi apparatus, specifically in proliferative neural progenitor cells. The average Oct4 intensity was calculated for cells that were inside and outside the clusters displaying enhanced Cx43 at 24 h (**d**). Cells within the Cx43-enhanced clusters at 24 h exhibited a significant lower Oct4 expression compared to cells outside of the cluster with low Cx43 signal. Scale bar: 30 µm

The gap junctional intercellular network has been implicated as a regulator of spatial patterning during the development of neural networks^8,27^. As such, we examined whether RA treatment initiated spatial remodeling of the intercellular network by assessing the membrane localization of connexin43 (Cx43), the primary connexin isotype expressed by ESCs^28^. Cx43 expression in untreated colonies (0 h) was concentrated at the cell-cell interfaces, usually as a series of punctate spots (Fig. 2a). In contrast, mitotic cells typically had diffuse Cx43 spread across their membrane that was not limited to cellular interfaces (Supplementary Figure 2). This diffuse Cx43 ‘ring’ pattern has previously been found to coincide with mitosis-specific phosphorylation of Cx43 that causes decreased plaque formation^29^. After 24 h, distinct clusters of cells with enhanced Cx43 staining between cells were observed (Fig. 2b). Each Cx43-enhanced cluster also showed diminished Oct4 expression (Fig. 2d), implying that the loss of pluripotency is concomitant with an increase in intercellular communication. After 24 h, distinct clusters of cells with enhanced Cx43 staining between cells were observed (Fig. 2b). Each Cx43-enhanced cluster also showed diminished Oct4 expression, implying that the loss of pluripotency is concomitant with an increase in intercellular communication. A similar increase in Cx43 signal was apparent in clusters of differentiated cells after 48 h, but with fewer cells per cluster compared to the 24-hour time point, indicating that the change was transient (Fig. 2c). Furthermore, the clusters at 48 h also displayed an accumulation of Cx43 in the cytoplasm. Interestingly, a similar accumulation of Cx43 is present in the Golgi apparatus of proliferative neural progenitor cells that is temporarily lost during differentiation^30^. Thus, ESCs exhibit a transient increase in Cx43 localization to their membranes as they undergo differentiation towards a neural progenitor state in response to RA-treatment.

### Intercellular transport as a function of cell cycle state

**Fig. 3:**
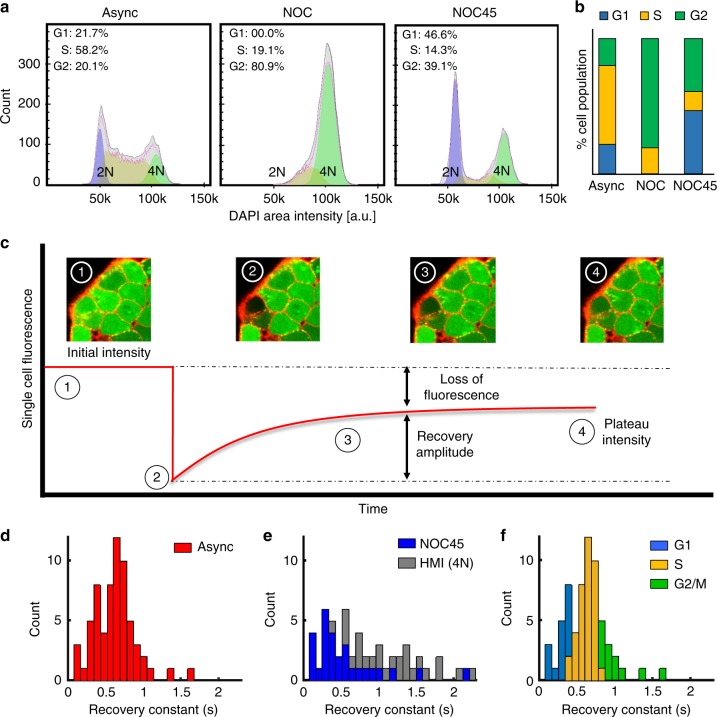
Analysis of intercellular transport rates as a function of cell-cycle state. **a** Asynchronous cell population (Async) shifted to G2-phase after nocodazole treatment (NOC), and after 45 min of recovery (NOC45) the population shifted to G1-phase. **b** The population distribution averages from **a** were calculated for the Async, NOC, and NOC45 treatment conditions. **c** An illustration of the GAP-FRAP technique for quantification of relative diffusion rates between adjacent cells. **d** A histogram of recovery time constants collected using gap-FRAP in the Async population (*n* = 64), where high and low recovery constants represent slow and fast transport rates, respectively. **e** The distributions of recovery constants for Hoechst-identified mitotic (HMI) cells (*n* = 26) and NOC45 (*n* = 28) were shifted to the right and left of the Async population, respectively, indicating slower and faster transport in these populations. **f** A projection of each cell cycle state onto the Async distribution using information from **a** and **e**, as described in Supplementary Figure 3

Given the capability of the cell cycle to modulate GJ connectivity and the rapid cycling time of pluripotent cells, we hypothesized that the cell cycle was a source of dynamic heterogeneity within the pluripotent-intercellular network. To characterize the degree of influence, intercellular transport rates were quantified at each stage of the cell cycle. The various cell cycle stages were distinguished by treatment with nocodazole to cause cell cycle arrest at G2/M-phase followed by the removal of nocodazole and subsequent recovery^31^. Sampling before (Async), immediately after (NOC), and 45 min after nocodazole (NOC45) treatment yielded three conditions: an asynchronous population, a synchronized population of G2/M-phase cells, and a recovered population containing both G1- and G2/M-phase cells (Fig. 3a, b). While NOC treatment is very efficient at arresting cells in G2/M-phase, the depolymerization of microtubules can affect other cellular processes. To minimize these influences, Hoechst-mediated identification (HMI) was adopted for quantification of M-phase cells in lieu of the NOC condition (Supplementary Figure 3). Hoechst treatment enabled M-phase cells to be visually distinguished by their 4 N nuclei configurations and provided an additional level of spatial data that was more representative of communication in the naturally asynchronous multicellular environment. The GAP-FRAP technique (detailed in Methods) was used to establish distributions of intercellular transport rates from the three conditions: Async, HMI, and NOC45. Briefly, GAP-FRAP monitors the diffusive transport of an intracellular fluorophore (calcein) into a single photobleached cell from neighboring interconnected cells (Fig. 3c)^32^. Recovery constants were mathematically derived using the perturbation-relaxation equation described in^32^ and have an inverse relationship with the functional transport rate of a cell.

The Async population yielded a unimodal distribution of recovery rates, predominantly representing S-phase cells (~60%)(Fig. 3a, d). The HMI population had significantly larger recovery constants on average (~50%, *t-*test: *p* < 1e-5) than that of the Async population, indicating slower transport for mitotic cells which is consistent with the literature^29^. In contrast, the NOC45 population primarily exhibited low recovery constants and fast intercellular transport (Fig. 3e). Since the NOC45 population consists mostly of G1- and G2/M-phase cells (Fig. 3b) and analysis of the HMI population showed mitotic cells with slow transport, it was deduced that G1-phase was associated with the observed fast transport. As a secondary validation, morphological analysis of the NOC45 tested cells showed that the rounded morphology typical of M-phase cells accompanied each case of slow transport in the distribution (Supplementary Figure 4). Therefore, by establishing that G1-phase and G2/M-phase produce fast and slow transport rates, respectively, the asynchronous distribution could be divided according to the proportion of each cell cycle state (Fig. 3b, f and Supplementary Figure 3).

### Cell-cycle dynamics generate intercellular heterogeneity

**Fig. 4:**
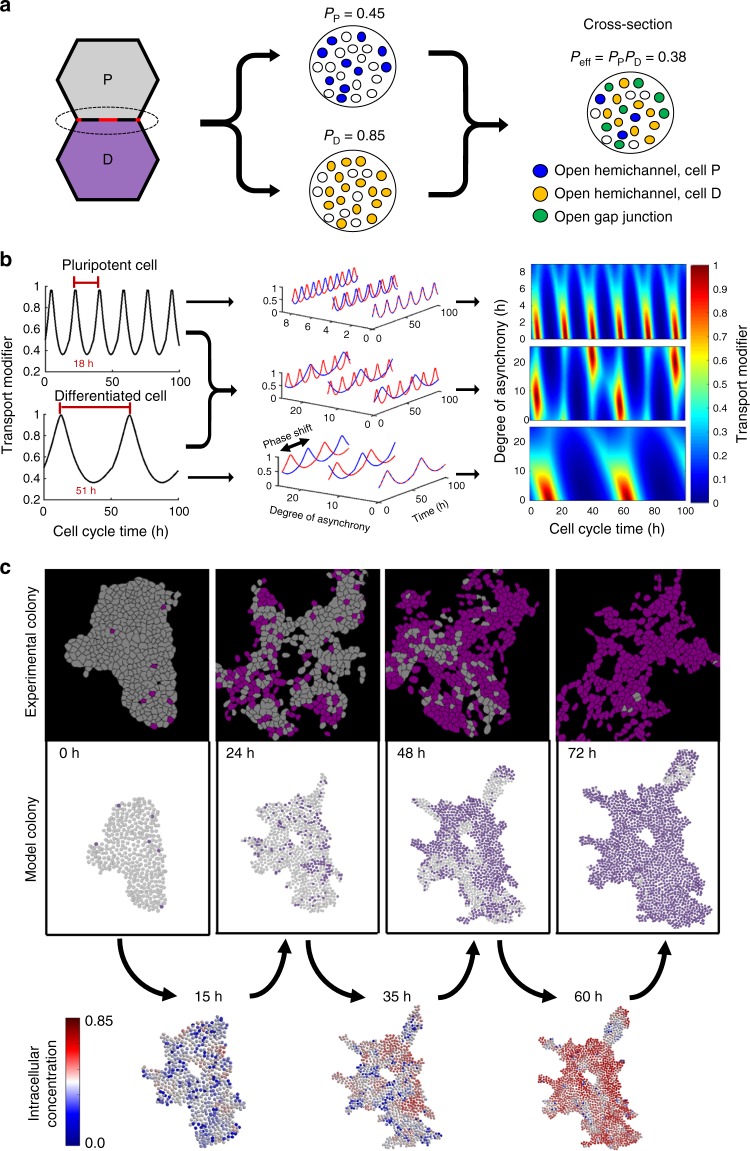
Computational analysis of cell-cycle modulation on intercellular communication. **a** Each cell type (P, pluripotent; d, differentiated) has a base permeability, representing the average percent of gap junction hemichannels open at a cell-cell interface, with the total percent of open channels being the product of the two base permeabilities. **b** A function for cell-cycle modulation over time was defined based on the transport trends noted in Fig. 3, with a convolution of all possible transport profiles that two cells could experience over time for P-P, P-D, and D-D. **c** The intercellular transport model was implemented within an agent-based model and compared to digitized experimental colonies over time. Supplementary Movie 1 is a representative video of the progression of intracellular gradient formation and differentiation

To further understand the collective effect of cell cycle heterogeneity on spatial patterning, we implemented intercellular diffusion as a conduit for initiating differentiation into an agent-based computational model in which individual cells divide, differentiate, and modulate their connectivity to neighboring cells according to state-specific rules^33^. The overall permeability between two cells was defined as the product of the individual base permeability (PM_n_) of each cell, reflecting the underlying biological mechanism of gap junction channel formation from neighboring connexon hemichannels^34^. To assess phenotypic differences in permeability, GAP-FRAP was performed on randomly selected cells within the differentiating populations after 24 and 48 h of RA exposure. In agreement with the increased Cx43 signal detected after RA-treatment (Fig. 2), there was a significant increase in the intercellular transport at both time points (Supplementary Figure 5). As such, a higher base permeability was assigned to differentiated cells in the model (Fig. 4a). The base permeability of each cell type was modulated by cell cycle state, with G1-phase having the highest permeability (100% PM_n_) and mitotic cells having the lowest (30% PM_n_, calculated from the ratio of average HMI to NOC45 recovery constants). The modulation function (Fig. 4b) was defined such that the residence time of fast and slow transport is proportional to the distribution of G1- and G2/M-phase cells in the asynchronous distribution (Fig. 3f). The function was scaled for each cell type based on cell cycle lengths previously established for Oct4 + and Oct4- cells^33^, with the caveat that RA is capable of inhibiting cell growth^35^.

Differentiation was initiated via two means: a) through a stochastic process, or b) when a small (<1 kDa), diffusible molecule accumulated within a cell past a specified threshold. The small molecule was assumed to be produced by both cell types and to be capable of intercellular transport via concentration gradients. A higher production rate of the small molecule was assigned to differentiated cells to reflect a possible neuro-inductive positive feedback in their local environment. The variability in transport potential was extensive with these differentiation and intercellular transport mechanisms implemented in the model. Unique intercellular transport profiles were produced as a function of time from cells dynamically interacting with adjacent cells at various stages of the cell cycle and differentiation states (Fig. 4b, Supplementary Movie 1). In contrast, a nearly uniform intracellular distribution within the population occurred when cell cycle was synchronized in the model, followed by rapid differentiation with no pattern formation (Supplementary Movie 2). While this result is exaggerated by the model constraints (i.e. intercellular communication as the primary differentiation mechanism), it emphasizes the role of the cell cycle in producing intercellular heterogeneity within cell populations. Indeed, a cell cycle gating mechanism is sufficient for generating a dynamic intercellular network that is capable of pattern formation.

### Intercellular diffusion generates differentiation patterns

The structure of the intercellular gap junction network rapidly established a non-uniform distribution of the small diffusing molecule in the model, starting from a relatively homogenous population of pluripotent cells randomly distributed throughout different phases of the cell cycle in the same ratio as an asynchronous population. Differentiation began in regions of the cellular network that accumulated high levels of the small diffusing molecule. The differentiated cells further modulated the intercellular network by having longer division times, thus longer G1- and S-phases, and instigated future differentiation patterns. Visually, the differentiation patterns produced by the model appeared similar to those observed in an experimental data set of time-lapse digitized images of Oct4 immunofluorescence (Fig. 4c). To quantitatively compare similarities in the spatial patterning, representative metrics were extracted from both experimental and model data for dimension reduction by principal component analysis (PCA).

**Fig. 5:**
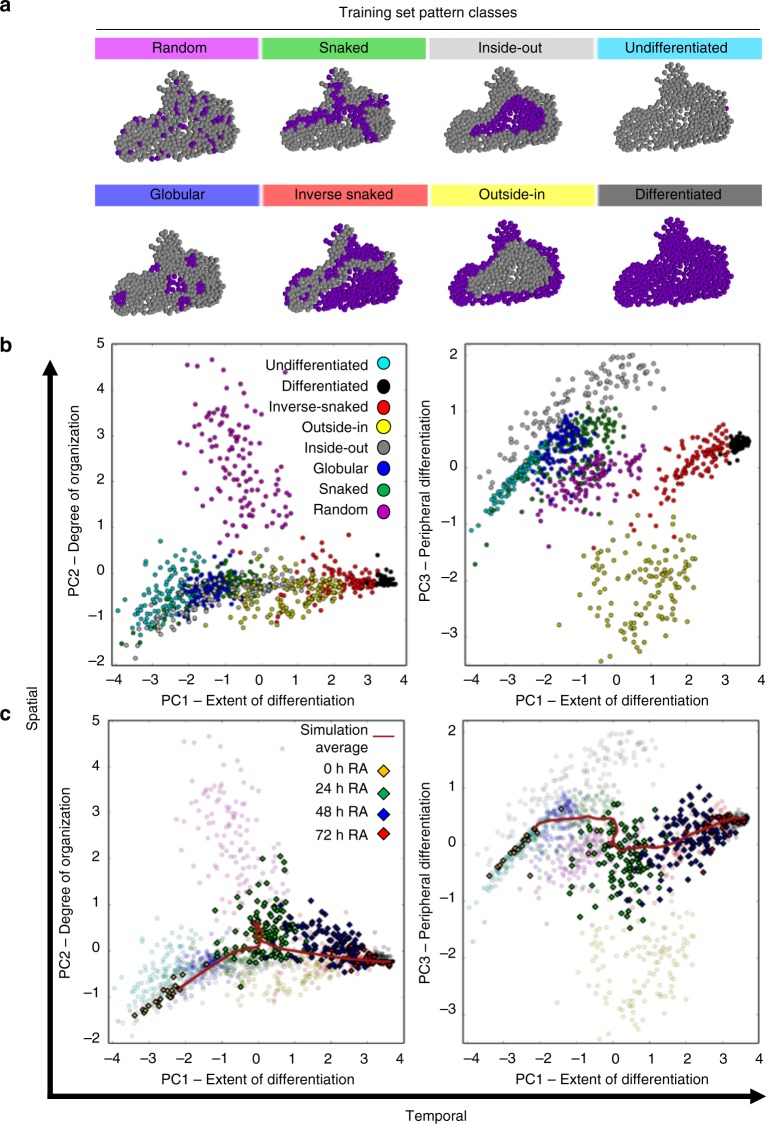
Quantification of spatial patterning during RA differentiation. **a** The computationally generated pattern class structures used to train the principle component analysis, derived from^33^, were applied to 120 experimental colony structures. **b** The seven selected metrics were calculated from each of the training set pattern classes (8 classes x 120 colony structures) and transformed into latent variable space through principal component analysis. PC1 represents extent of differentiation (temporal), and PC2 and PC3 represent organization/stochasticity and spatial locale, respectively (spatial characteristics). **c** The same metrics were calculated from experimental images of 0- (*n* = 24), 24- (*n* = 113), 48- (*n* = 139), and 72-h (*n* = 22) RA-treated colonies and transformed into latent variable space. The average simulation trajectory was capable of capturing the spatiotemporal trajectory of the experimental data (see Supplementary Figure 7 for simulation data points). At 24 h there is a steep transition along both spatial axes, indicating that there is a gain in random differentiation and that it propagates along the edges of colonies. By 48 h, the majority of differentiated cells are connected within a single, asymmetrical cluster

A training set of 2D patterns classes that occur during ESC differentiation were computationally produced for pattern classification and to define descriptive metrics (Fig. 5a)^33^. An original set of 15 potential network metrics, where each metric represented a physical or spatial characteristic of the colony, were calculated from the 960 computationally defined patterns (8 patterns x 120 colony structures). PCA was performed on the set of network metrics, condensing the multivariate characterization of each pattern class into latent variables and conferring a unique ability to visualize similarities and relationships between the patterns. Seven of the metrics were found to be the most capable of separating the various pattern classes into discrete clusters when plotting the principal component (PC) loadings (Fig. 5b, Supplementary Figure 6). Furthermore, the three PCs derived from the seven selected metrics each represent a particular property of the differentiation within a colony. Specifically, PC1 represents the extent of differentiation, with each pattern effectively being separated by the percent of differentiated cells in the colony. PC2 reflects the relative spatial organization, ranging from the Random pattern class to highly organized, single cluster patterns, such as Inside-Out and Outside-In. PC3 illustrates the locale of differentiation, or whether Oct4- cells tend to cluster on the periphery of a colony or in central regions with high cell density. The first three components accounted for ~88% of data variance (PC1: 59%, PC2: 15%, PC3: 14%) and provided a quantitative means of describing the spatiotemporal properties of differentiating cell populations.

The previously mentioned time-lapse images and simulation data of differentiating cell populations were converted to latent space by applying the trained-PCA transform, which is an effective means for analyzing dynamic morphogenic trajectories (Fig. 5c) as we have previously reported^33,36^. Our experimental data exhibited a temporal trajectory as it transitioned from an undifferentiated to a differentiated fate along PC1. Although there was large variability in patterning at 24 h along PC2, with colonies spread across multiple pattern classes, the simulation data indicated that this phenomena represented a transitional state in the spatial organization of differentiation. Specifically, each simulation showed a slow accumulation of spatially heterogeneous differentiation within the population followed by a fast differentiation event, occurring ~24 h, that forms clusters of differentiated cells. Differentiation preferentially occurred along the edges of colonies, as indicated by the data and trajectory approaching the Outside-In pattern class (Fig. 5c, PC3). In addition to differentiation initially advancing along the outside of a colony, loss of Oct4 also tended to propagate between differentiated cells. The manifestation of this behavior became apparent at 48 h where nearly every differentiated cell was connected as a single cluster. For example, in Fig. 4c the edges of the experimental colony are almost entirely differentiated and strands of differentiated cells form through the center to connect the edges. A similar pattern occurs within the intercellular diffusion model where paths of high concentration form and propagate differentiation between already differentiated cells. Thus, our model was able to accurately recapitulate the trajectory of differentiation using the intercellular network to inform cell fate decisions in a spatially organized fashion.

### Intercellular perturbation modulates rate of differentiation

Nocodazole synchronization was originally proposed as a fundamental perturbation to test the predictive capability of the intercellular differentiation module. However, this proved to be technically unfeasible since cell cycle asynchrony is regained rapidly after short-term nocodazole treatment and long-term nocodazole treatment decreases the expression of pluripotency markers at a similar time scale as RA treatment^37^. Instead, we inhibited the production of an abundant small molecule that could be represented by the generic diffusible molecule in the computational model. Cyclic AMP (cAMP) is a canonical secondary messenger that has been directly associated with creating spatially diverse intracellular distributions as a function of intercellular communication^13^. We hypothesized that the distribution of cAMP plays a role in the spatial aspects of RA-accelerated differentiation since RA treatment can activate PKA, a cAMP dependent kinase^38^. While cAMP is involved in multiple cellular processes, the availability of reagents to perturb intracellular cAMP levels makes it an attractive molecular target for investigating GJ-mediated communication and testing the validity of our model. Adenylyl cyclase (AC) was inhibited with SQ22536 (SQ) to prevent the conversion of ATP to cAMP, and consequently decrease the intracellular cAMP concentration^39^.

**Fig. 6:**
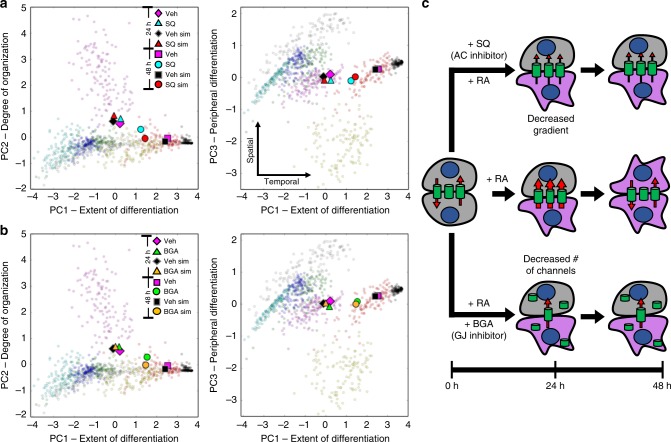
Perturbation to the intercellular network of a multicellular D3 ESC population affects RA-accelerated differentiation in a temporal manner. At 24 h, neither adenylyl cyclase (AC) inhibition (**a**, SQ-treatment) nor gap junction (GJ) inhibition (**b**, β-GA-treatment) induced a significant change in spatial or temporal characteristics of differentiation compared to the vehicle control. By 48 h, a temporal shift along PC1 is observed for both treatments (**a**, **b**), depicting a decrease in the rate of differentiation. The intercellular model accurately predicted these dynamics, represented by average values for SQ-treatment (Exp: *n* = 87, Sim: *n* = 30) and BGA-treatment (Exp: *n* = 62, Sim: *n* = 30) compared to the vehicle control (Exp: *n* = 87, Sim: *n* = 75) (**a**, **b**). A schematic diagram of our proposed mechanism for the influence of AC and GJ inhibition on differentiation potential is depicted in **c**. Specifically, both AC and GJ inhibition are suggested to decrease the intercellular flux between cells but via separate mechanisms: modulating the concentration gradient and the number of open channels for AC and GJ inhibition, respectively. Data clouds of **a**, **b** before averaging are shown in Supplementary Figures 8, 9

The stochasticity and peripheral differentiation in SQ-treated colonies increased slightly at 24 h compared to the vehicle DMSO control. Furthermore, SQ-treated colonies showed significantly delayed differentiation between 24 and 48 h, retaining a large proportion of pluripotent cells. The simulation of SQ-treatment in the model, produced by decreasing the cAMP production rate of differentiated cells, resulted in similar dynamic changes and comparable distributions of spatial patterns at 24 and 48 h relative to the experimental data (Fig. 6a).

To test the degree of influence from the intercellular network, we investigated the effect of gap junction inhibition during differentiation. Simulations of GJ inhibition yielded a delay in the differentiation rate and minimal variation in the spatial organization or locale of differentiation, comparable to the results from inhibiting cAMP conversion. To test this computational prediction, we inhibited gap junctions with β-glycyrrhetinic acid (β-GA), a small molecule that causes dephosphorylation of connexins and disassembly of gap junctions^40^. The potent non-selective inhibition of GJ channels by β-GA results in almost complete abrogation of intercellular communication, with the caveat that β-GA can increase mineralocorticoid activity via 11 β-hydroxysteroid dehydrogenase inhibition. However, mineralocorticoid receptor expression is low in murine ESCs and is unlikely to impact early neural commitment^41^. The experimental perturbation to GJ-channels by β-GA resembled the outcome predicted by the simulations, with differentiation delayed at 48 h in a manner similar to, but less potent than, AC-inhibition (Fig. 6b). Collectively, these results lead to several conclusions: (i) delaying differentiation by SQ-treatment implies that cAMP has a role in stimulating neural differentiation during RA-treatment; (ii) the ability of the model to capture the temporal change indicates that this role is at least partially mediated by the intercellular network; and iii) the minor spatial changes upon perturbation reflect that the topology of the gap junction network remains the same despite the slower differentiation rate.

As further validation, both perturbations were applied to ATCC G-Olig2 cells, derived from the RW4 ESC line (Supplementary Figures 1,0–1,2). In both conditions, the temporal delay was recapitulated, with β-GA having a slightly more significant effect than the SQ-treatment. Interestingly, the intercellular model was able to accurately predict each condition by from the 19 -and 43-hour time points of the D3 simulations, indicating that the RW4 cells are inherently either more pluripotent than D3 cells or that they are less sensitive to RA-treatment. A few other notable differences between the cell lines became apparent in our analysis. For instance, the RW4 colonies appeared to be less specific for initiating differentiation along colony edges. However, the propagation of differentiation between clusters remained a prominent spatial feature, as demonstrated by the frequent emergence of the ‘Snaked’ pattern class (Supplementary Figure 13a). Also, GJ inhibition by β-GA induced a pronounced morphological change in RW4 colonies at the 48 h time point (Supplementary Figure 13b). Specifically, a web-like pattern consisting of strands that were 1–2 cells thick formed and was interpreted in latent space as an increase in stochasticity.

The similarity in response to the different inhibition mechanisms can be attributed to the decreased intracellular accumulation of a pro-differentiation molecule caused by limiting the intercellular transport, either by decreasing the number of active channels (β-GA) or by decreasing the gradient between cells (SQ) (Fig. 6c). Furthermore, the consistency in the temporal shift between the two ESC lines from both inhibitor treatments highlights the general principle of intercellular communication mediating the propagation of differentiation signals.

## Discussion

In this work, we characterize the spatial organization that occurs during early neural differentiation of pluripotent mESC colonies. Differentiation initiates a redistribution of Cx43 within the population and increased connectivity between differentiating cells. In addition to phenotypic changes, intercellular heterogeneity is modulated by cell cycle state. The pro-differentiation molecular gradients driving intercellular transport, as a function of cell cycle and phenotype, result in dynamic Oct4 patterning in our computational model that recapitulates the spatiotemporal trajectory of differentiation during early neural commitment. Furthermore, we find that perturbing intercellular communication by disruption of GJ formation or inhibiting levels of a critical molecule transported via GJCs can delay the propagation of differentiation, indicating a vital role in how differentiation processes can be controlled for design of multicellular systems.

We identified that the cell cycle is an important regulator of intercellular heterogeneity within pluripotent multicellular systems and necessary for the emergence of colony-wide patterning. During mitosis, a distinct phosphorylation state of Cx43 exists (Cx43-P_m_) that is not incorporated into gap junction plaques. The decrease in plaque formation corresponds with a reduction in connectivity and transport between mitotic and non-mitotic cells^29^. While no direct link has been made between G1-phase and increased intercellular transport, cyclin-D1 and Cx43 expression have been correlated^42^. The increased transport during G1-phase might result from a concurrent peak in cyclin-D1 and Cx43 expression as a function of MEK-ERK activation. The MEK-ERK pathway has been shown to activate Cx43 expression and is the primary activator of cyclin-D1 expression during G1-phase^43^. However, the correlation between cyclin-D1 and Cx43 is likely more complex than a single pathway, as there have been conflicting reports on MEK/ERK activation of Cx43^44–46^. While identifying the mechanism is beyond the scope of this study, the modulation of intercellular transport during cell cycle progression has a considerable impact on the formation of intracellular gradients. As shown by our model, vast intercellular heterogeneity can be generated by the natural asynchrony of the cell cycle, even within relatively homogenous populations of cells.

We find that the intercellular network gains complexity as neural commitment occurs. Most cell types express a unique profile of connexin isotypes and, as differentiation occurs, both the composition and proportion of Cxs within the cell can change. Furthermore, Cx isotypes each have distinct permeabilities for diffusing molecules and capacity for forming channels with other isotypes. The result is an intercellular network capable of phenotypic-compartmentalization, opposing gradients of small molecules, and even unidirectional transport^11,12^. Also, most Cxs are susceptible to post-translational modifications that affect GJ formation, degradation, and permeability^47^. As such, intercellular transport can be modulated during differentiation both as a function of transcription and Cx activity. The rapid and sequential gain and loss of Cx43 membrane localization between 24 and 48 h of RA treatment suggests that the increased connectivity in Oct4-diminished clusters was predominantly due to a redistribution rather than a change in expression of Cx43. We hypothesize that enhanced assembly of Cx43 via cAMP/PKA activation, which has been linked with RA treatment, produced the redistribution^38^. In terms of the cytoplasmic accumulation of Cx43, the locale and staining pattern agrees with previous studies demonstrating localization in the Golgi apparatus^30^. Since Cx43 connexons are formed in the Golgi network before being trafficked to the membrane^30,47^, an accumulation in the Golgi network would agree with enhanced assembly. Despite the decreased number of cells at 48 h displaying enhanced Cx43 signal, a faster intercellular transport rate was maintained compared to pluripotent cells (Supplementary Figure 5). Therefore, it is possible that either Oct4- cells transcribe a separate Cx isotype or that the prolonged cell cycle associated with differentiation masks the decreased Cx43 expression relative to the Oct4-diminished cells. Specifically, the extended G1/S-phase relative to M-phase associated with differentiation would also cause the mean transport rate to be faster.

Additionally, inhibition of cAMP may suppress the PKA activation associated with RA treatment, preventing the enhanced assembly of Cx43. The effect of decreased cAMP production due to AC-inhibition could thus reflect an indirect perturbation to the intercellular network rather than a direct impact of cAMP on differentiation potential (Fig. 6b). Consequently, cAMP would act as a concentration-sensitive modulator of intercellular connectivity, where cAMP levels above a specific threshold cause increased transport. Therefore, the intercellular network is not only affected by transcriptional changes that occur during differentiation, but is also responsive to changes in the intracellular environment.

We developed a multicellular model that describes cell fate transitions coupled with intercellular communication that is regulated by the dynamics of connexins as a function of the cell cycle. Although previous computational models of intercellular communication have included the biophysics of gap junction transport, they have primarily focused on small molecule diffusion through individual channels^48,49^. One notable multicellular model that focused on ion transfer relied upon membrane potential to regulate intercellular connectivity^50^. While capable of examining the development of intracellular gradients at a multicellular level, the bioelectric model is constrained to a static system. A primary advantage of our selected framework is its ability to investigate the impact of dynamic intercellular topologies on a multicellular system that includes growth, division, and differentiation. The dynamic network facilitates the study of evolving spatial organization and the resultant effect on the formation of intracellular gradients.

Interestingly, a recent paper proposed an alternative, hidden Markov model framework that explored the role of stochasticity during early neural differentiation, but without consideration of spatial organization and cell-cell communication^51^. While the mechanistic framework is different from the work presented here, they share several similarities. In particular, both models consider differentiation to be an autonomous process with discrete state changes that are probabilistic. The probability of a state transition in our model, however, is dependent on the accumulation of an intracellular metabolite within a cell rather than the purely stochastic progression through microstates. The description of microstates typically considers gene and protein expression patterns, but in the present work the metabolic profile of a cell also impacts susceptibility to differentiation signals^52,53^. The connection between microstates and the intracellular accumulation of metabolites is further highlighted by the temporal delay effect of both AC- and GJ-inhibition on the trajectory of differentiation (i.e. slower microstate progression). The cellular communication integrated within our model via gap junction-mediated transport leverages both stochastic state transitions (consistent with Stumpf et al.) and the higher-order collective behavior of the multicellular system, suggested by our observations of pattern propagation within pluripotent colonies. Therefore, our model both complements this prior modeling study while also advancing the description of spatial organization effects on the emergence and propagation of micro- and macrostates during early neural differentiation.

The superposition in latent variable space between experimentally derived results and agent-based simulations is a powerful feature of the analysis pipeline presented here. The extraction of network metrics is particularly necessary for the accurate quantification of systems with variable and asymmetrical structures, such as during unconstrained monolayer growth (Fig. 4c). Since each colony structure can have a unique configuration, the spatial characteristics used to distinguish pattern classes need to be independent from the underlying morphology. Network metrics accomplish this by evaluating relationships between neighboring nodes/cells and colony-wide. Furthermore, the transformation of each multivariate set of metrics to latent space enables visual and numerical comparison between individual colonies. The training set of artificially generated colony patterns provides the ability to directly map regions of latent space to specific spatial organizations (e.g. the “random” cloud versus a highly organized “outside-in” or “inside-out” cloud). Accordingly, transformed sets of colony metrics from both experimental and computational sources can be delineated according to their proximity to each mapped pattern class in latent space. Computational models therefore can be designed to replicate morphogenic trajectories in a quantifiable manner and elucidate the factors that influence specific pattern formations.

In summary, this work demonstrates that intercellular heterogeneity can be generated by dynamic fluctuations in GJ permeability via the cell cycle. We found that the spatial trajectory of early neural differentiation is remarkably robust to perturbation through small molecule inhibition, but that the temporal progression of differentiation can be modulated. Ultimately, identifying the effect of molecular regulators of differentiation at a multicellular scale is an initial step towards replicating morphogenic events in vitro and successfully directing the emergence of desired engineered multicellular structures.

## Methods

### Cell culture

The murine embryonic stem cell line (D3) was cultured at 37 °C in monolayer on 100-mm tissue culture plates coated with 0.1% gelatin (Millipore EmbryoMax) in Dulbecco’s modified Eagle’s medium (DMEM) supplemented with 15% fetal bovine serum (FBS) (Atlanta Biologicals, Atlanta, GA), 2 mM L-glutamine (Lonza), 100 U mL^−1^ penicillin, 100 μg mL^−1^ streptomycin, and 0.25 μg mL^−1^ amphotericin (MP Biomedicals), 1x MEM nonessential amino acid solution (Corning), 0.1 mM 2-mercaptoethanol (Sigma-Aldrich), and 10^3^  mL^−1^ leukemia inhibitory factor (LIF) (EMD Millipore). Cells were passaged every 2–3 days, using 0.05% trypsin (Corning) to dissociate cells, centrifuged at 200 rcf for 5 min, and plated at a density of 20000 cells cm^−2^.

### Cell lines

ES-D3[D3] (ATCC® CRL-1934™)

G-Olig2 (ATCC® SCRC-1037™)

A MycoAlert™ PLUS Mycoplasma Detection Kit (Lonza LT07-703) was used to verify that there was no mycoplasma contamination in our cultures.

### Differentiation protocol

Cells were plated at a density of 10000 cells cm^−2^on Ibidi μ-slides coated with a 0.1% gelatin, 0.05% fibronectin solution. Cells were allowed to grow under regular cell culture conditions described earlier for 24 h before being transitioned to N2B27 medium containing 1 μM retinoic acid (Sigma-Aldrich). The N2B27 medium was composed of 50% DMEM/F12 (Thermo Fisher Scientific) and 50% Neurobasal Media (Thermo Fisher Scientific), supplemented with 0.5% N-2(100×) (Thermo Fisher Scientific) and 1% B-27(50×) (Thermo Fisher Scientific). Every 24 h, the medium was replaced with fresh N2B27 medium containing 1 μM retinoic acid.

### Immunofluorescence

For staining of Oct4 and Sox2, cells were fixed in 4% paraformaldehyde (Thermo Fisher Scientific) for 5 min and then permeabilized with 0.1% Triton X100 for 15 min. Samples were blocked with 2% normal donkey serum (NDS) (Sigma-Aldrich) for 1 hour and then incubated overnight at 4 °C with the following primary antibodies in 2% NDS: goat polyclonal Oct-3/4 (Santa Cruz, 1:200) and rabbit polyclonal Sox-2 (Thermo Fisher Scientific, 1:200). After washing, samples were incubated in a 2% NDS secondary antibody solution of donkey anti-goat Alexa Fluor 568 (Thermo Fisher Scientific, 1:200) and donkey anti-rabbit Alexa Fluor 647 (Thermo Fisher Scientific, 1:200) for 45 min before counterstaining with Hoechst 33342 (1:1000) in DI water. All images were collected on a PerkinElmer UltraVIEW VoX spinning-disk confocal microscope with a sCMOS camera at 20X magnification.

For Cx43 and Oct4 staining, the same procedure was utilized but with the following changes: The duration of fixation was increased to 15 min, the primary antibodies were goat polyclonal Oct-3/4 (Santa Cruz, 1:200) and rabbit polyclonal Cx43 (Sigma, 1:400), and the images were collected at 60x magnification. Catalogue numbers for antibodies are in Table 1.

**Table 1:** List of antibodies and their respective catalog numbers

Target	Company	Catalog #	Host
Oct-3/4 (N-19)	Santa Cruz	sc-8628	Goat
SOX2	Invitrogen	PA1-16968	Rabbit
Cx43	Sigma-Aldrich	C6219	Rabbit

### GAP-FRAP

To form colonies, mouse (m)ESCs were plated at a density of 10,000 cells cm^−2^ on Ibidi μ-slides, coated with a 0.1% gelatin, 0.05% fibronectin solution, for 24 h. A 1 mM calcein-AM (Thermo Fisher Scientific) stock solution in DMSO was diluted into 100 μL DMEM media and vortexed. The diluted calcein-AM solution was added to each well of the Ibidi μ-slide to a final concentration of 1 μM for 40 min at 37 °C. After 40 min, Alexa Fluor 647–conjugated wheat germ agglutinin (WGA) (Thermo Fisher Scientific) was added to each well to a final concentration of 10 μg mL^−1^, for 5 min at 37 °C. Each well was rinsed once before phenol red–free DMEM (Corning) medium was added for imaging. Images were acquired with a Zeiss NLO 710 confocal microscope, using built in software for photobleaching individual cells. Bleaching was initiated after acquiring three images, then maximum laser intensity was pulsed for 30 iterations before measuring fluorescence intensity in the photobleached cell every 3.8 sec. For identifying mitotic cells, Hoechst 33342 was added with the WGA for 5 min at a 1:1000 dilution. When quantifying nocodazole-treated cells, each well was treated with 100 ng mL^−1^ of nocodazole (Sigma-Aldrich) for 4 h before replacing with new DMEM medium containing 1 μM calcein-AM and incubating for 40 min at 37 °C, followed by 5 min with WGA, then changing to phenol red–free DMEM for imaging.

From each GAP-FRAP experiment, the measured fluorescence intensity value in the photobleached area was compiled as a function of time, setting t = 0 as the time point with the minimum intensity value after bleaching. The intensity values were normalized to the maximum intensity after bleaching and fitted to an exponential function of the form $I = 1 - {\mathrm{exp}}\left( { - \frac{t}{{R_{\mathrm{c}}}}} \right)$ where *R*_c_ is the recovery constant. The recovery constant was then normalized to the maximum fluorescence intensity before bleaching. In a few cases, it was also possible to quantify the recovery lost from neighboring donor cells (Supplementary Figures 1, 4). However, the context of the intercellular transport is different for the neighboring cells compared to the photobleached cell. Specifically, the photobleached cell only experiences a net influx of unbleached calcein, whereas the neighboring cells have both a loss of calcein to the photobleached cell and an influx from secondary neighbors. As such, measurements from secondary cells are not included in any of the distributions for characterizing transport rates in Fig. 3.

### Flow cytometry

For quantifying differentiation progression, cells were grown for 24 h on 100 mm tissue culture plates coated with 0.1% gelatin. Each plate was rinsed with PBS then differentiated using our previously stated differentiation protocol, with samples taken at 0, 24, 48, and 72 h from the start of the protocol. Each sample was dissociated using 0.05% trypsin, centrifuged for 5 min at 200 rcf, and fixed using 4% paraformaldehyde for 10 min. Cells were permeabilized by centrifuging at 200 rcf with 5% Triton X100 for 5 min. To prevent non-specific binding, cells were incubated for an hour in 10% NDS before being treated with primary antibodies Oct-3/4 (Santa Cruz, 1:200) and Sox-2 (Thermo Fisher Scientific, 1:200) in 10% NDS for 1 h. Cells were washed 3 times then incubated for 30 min in 10% NDS containing the following secondary antibodies: Donkey anti-goat Alexa Fluor 488 (Thermo Fisher Scientific, 1:200) and Donkey anti-rabbit Alexa Fluor 647 (Thermo Fisher Scientific, 1:200).

For cell cycle analysis, the same protocol was used but with incubation in Hoechst 33342 (1:1000) for 15 min instead of primary and secondary antibody staining. All flow cytometry measurements were collected on a BD LSR Fortessa flow cytometer.

### Agent-based model

An agent-based model was developed for simulation of pluripotent colonies by refining a python-based custom platform first described in White et al^33^. Here, each cellular agent with a set of properties of cell radius, spatial coordinates, division time, cell type, and intracellular concentration. Cell radius was set at 6.5 µm based on previous data collected in our lab for D3 cells, with cells modeled as rigid bodies. Spatial coordinates were initially set using a digitized set of coordinates from an experimental colony, assuming 100% pluripotent cells. Division time is randomly distributed between 0 and 18 h for each cell and is defined as the time since the cell last divided, with pluripotent cells dividing every 18 h and differentiated cells dividing every 51 h. As the colony grows it is restricted to a 2D-plane, with a collision detection algorithm optimizing cellular movement to prevent overlap of cells in the model. The asymmetrical growth of the colony is accomplished by preferential movement of cells away from the central mass of the colony when resolving overlap during collision detection. The initial intracellular concentration of each cell is randomly selected between 0.6 and 0.65, with a nascent permeability, production rate, and degradation rate specific to each cell type (Pluripotent, Differentiated). Table 2 presents parameter values for each cell type.

**Table 2:** Cell type specific parameters used in ABM of intercellular communication

Cell type	Pluripotent	Differentiated
Cell radius (*r*)	6.5	6.5
Cell cycle length (CCL)	18 h	51 h
Nascent permeability (PM_n_)	0.45	0.85
Production constant (vp)	4.89E-6	6.0E-6
*K*_50_	0.03	0.04
Degradation constant (Kd)	1.3E-7	1.3E-7

The intercellular network is determined at each time step by calculating the intercellular distance between every cell, and if the cell membranes are within 2 microns (2xradius + 2 um) then they are “connected”. Since the resistance to diffusion is significantly higher at the gap junction interface, we consider the intracellular compartment to have instantaneous mixing after every diffusion time step. The flux between each cell follows Fick’s first law, accounting for the individual permeability of the connected cells (function of cell cycle and cell type, Eq. 1–6.

*t*_Div_: Current division time, between 0 and CCL for current cell

PM_max_: Maximum permeability, the maximum flux between any two cells if all gap junctions were open

1 $${\mathrm{DT}}_{{\mathrm{norm}}} = {\mathrm{Abs}}\left( {{{t}}_{{\mathrm{Div}}} - \left( {\frac{{{\mathrm{CCL}}}}{4}} \right)} \right)$$

DT_norm_ is a transform function, shifting the minimum value forward by ¼ of the cell-cycle length (Supplementary Figure 1,5). This shift decreases the steep transition in cell-cycle time that is seen in *t*_div_ when a cell starts a new division cycle (@18 h simulation time), while maintaining individual regions of minimum and maximum values per period.

2 $${\mathrm{Cell}}{\hskip2pt}{\mathrm{Cycle}}{\hskip2pt}{\mathrm{Effect}}\left( {{\mathrm{CCE}}} \right) = \frac{1}{{1 + \left( {\frac{{{\mathrm{DT}}_{{\mathrm{norm}}}}}{{{\mathrm{CCL}}}}} \right)^2}} + \frac{{0.69t_{{\mathrm{Div}}}^6}}{{{{t}}_{{\mathrm{Div}}}^6 + {\mathrm{CCL}}^6}}$$

The cell-cycle effect function is defined such that maximum transport would occur during the first few h of a new division cycle when a cell would be in G1-phase, followed by a decline throughout S-phase to reach a minimum transport value near the end of a division cycle when a cell would be undergoing mitosis.

3 $${\mathrm{PM}}_{{\mathrm{n},{\mathrm {eff}}}} = {\mathrm{PM}}_{{{\max}}} \left( {\mathrm{CCE}}_{{\mathrm{cell 1}}} \ast {\mathrm{PM}}_{{\mathrm{n}},{\mathrm{cell}}1} \right)\left( {\mathrm{CCE}}_{\mathrm{cell 2}} \ast {\mathrm{PM}}_{{\mathrm{n}},{\mathrm{cell 2}}} \right)$$

The effective permeability between any two cells is equal to the maximum permeability adjusted by the product of the nascent permeability of the adjacent cells. The nascent permeability is defined by cell type (differentiated or undifferentiated) and regulated by the Cell cycle effect function.

4 $${\mathrm{Prod}} = \frac{{{\mathrm{vp}}}}{{1 + {{C}}_{\mathrm{t}}/K_{50}}}$$

5 $${\mathrm{Deg}} = - k_{\mathrm{d}}C_{\mathrm{t}}$$

6 $${\mathrm{Flux}} = {\mathrm{PM}}_{{\mathrm{n}},{\mathrm{eff}}}\Delta C + {\mathrm{Prod}} + {\mathrm{Deg}}$$

Production, degradation, and flux in and out of a cell are calculated every 3 s, or 1200 times per differentiation time step, with a continuously updating intracellular concentration. For the production function, we use an inhibitory Hill function with an *n*-value of 1 to represent decreased production when intracellular concentrations are high. A simple degradation function is used with the degradation constant the same for both cell types. Diffusive flux between adjacent cells is calculated as the product of the effective permeability and the concentration gradient between each neighboring cell. The final concentration at the end of every hour is used to determine the probability of differentiation, as per Eq. 7.

7 $$P\left( {{\mathrm{diff}}} \right) = \frac{{{\mathrm{tC}}^{\mathrm{n}}}}{{{\mathrm{tC}}^{\mathrm{n}} + {{K}}_{{\mathrm{th}}}^{\mathrm{n}}}}$$

Threshold concentration (*C*_th_): 0.74

tC: Threshold count, number of timesteps a cell is over *C*_th_

$$\begin{array}{l}ie.{\mathrm{IF}}\,C > C_{\mathrm{th}}:{\mathrm{tC}} = {\mathrm{tC}} + 1\,{\mathrm{ELSE}}:{\mathrm{tC}} = {\mathrm{tC}} - 1\\ {{K}}_{{\mathrm{th}}} = 2\\ {{n}} = 4\end{array}$$

A stochastic differentiation term is incorporated to represent external factors affecting differentiation that are not mechanistically captured by our intercellular diffusion module. Since the main external factor was the addition of retinoic acid, which was replaced every 24 h, the stochastic term is made oscillatory to correspond with RA-addition. Specifically, differentiation potential increases for 12 h after RA-addition then decreases back to the base differentiation potential, with this repeating every 24 h (Eq. 8).

8 $$P\left( {{\mathrm{stochastic}}} \right) = 0.002 + {\mathrm{Abs}}\left[ {0.001 \ast {\mathrm{sin}}\left( {\frac{{t_{{\mathrm{sim}}}}}{8}} \right)} \right]$$

where *t*_sim_ is the simulation time.

### Image analysis

To convert our experimental images into digital networks, we used a CellProfiler (http://cellprofiler.org/) pipeline (Supplementary Figures 1, 6). The pipeline uses the blue channel (Hoechst) to identify nuclei within the image, and then uses propagation to define cellular boundaries. Both red and green channels, representing Sox2 and Oct4, respectively, first use a local MCT threshold to separate high expressers from low expressers and background noise. After thresholding, the red and green channels are converted to masks and applied to the cell objects identified using the blue channel. This data is exported to a CVS file and imported into python, where it is represented in an analogous format as our simulation data.

### Latent space analysis

We computationally generated 2D pattern classes defined for embryoid body differentiation. Each pattern class was produced using a previously digitized experimental network, resulting in 120 unique colony structures. To better distinguish spatially localized clusters, the differentiation status of a cell was also a function of the differentiation status of neighboring cells. Specifically, if the percent of differentiated neighbors is greater than the average number of differentiated neighbors for every cell in the population, it is classified as differentiated. From each network, seven metrics were extracted as represented in Supplementary Figure 6. After collecting these metrics for every pattern class generated in our training set (7 metrics × 960 networks), the data is mean centered, scaled to unit variance, then transformed using Principal Component Analysis (PCA). The same metrics were extracted from both simulation and experimental data, scaled/normalized in an identical fashion to our training set, and transformed into latent variable space using the previously trained PCA transform. The average trajectories for simulations were calculated by averaging every data point at a specific time.

### Statistics

All tests of significance, unless otherwise stated, were conducted via *t*-test with a significance level (*α*) of 0.01. Samples for the analysis of the differentiation trajectory and perturbation studies were collected from three different culture plates for each time and perturbation, with each plate contributing 10–20 images to the final *n*-values listed in their respective figure legends.

### Code availability

The computational model can be accessed from Github (DOI: 10.5281/zenodo.1413539) Software utilized: Python 2.7, CellProfiler 2.1.1

## Electronic supplementary material

Supplementary Information

Description of Additional Supplementary Files

Supplementary Movie 1

Supplementary Movie 2

## Data Availability

All data is available from the corresponding author upon request.
