# Author Correction: Dynamic intercellular transport modulates the spatial patterning of differentiation during early neural commitment

**DOI:** 10.1038/s41467-018-07442-0

**Published:** 2018-11-16

**Authors:** Chad M. Glen, Todd C. McDevitt, Melissa L. Kemp

**Affiliations:** 1 The Wallace H. Coulter Department of Biomedical Engineering, Georgia Institute of Technology and Emory University, Atlanta, GA 30332 USA; 2 Gladstone Institute of Cardiovascular Disease, San Francisco, CA 94158 USA; 3 Department of Bioengineering & Therapeutic Sciences, University of California, San Francisco, CA 94158 USA

Correction to: *Nature Communications*; 10.1038/s41467-018-06693-1; published online 05 October 2018

In the original version of this Article, an incorrect DOI number was provided in the Code Availability statement regarding the deposition of the computational model. The correct DOI is 10.5281/zenodo.1413539. This error has been corrected in both the PDF and HTML versions of the Article.

