# Supplementary material for: Dynamic intercellular transport modulates the spatial patterning of differentiation during early neural commitment
Source: Nat Commun. 2018 Oct 5;9:4111. doi: 10.1038/s41467-018-06693-1 (PMC6173785; doi:10.1038/s41467-018-06693-1)
Supplement: Supplementary file 1 — Supplementary Information [file 41467_2018_6693_MOESM1_ESM.pdf]

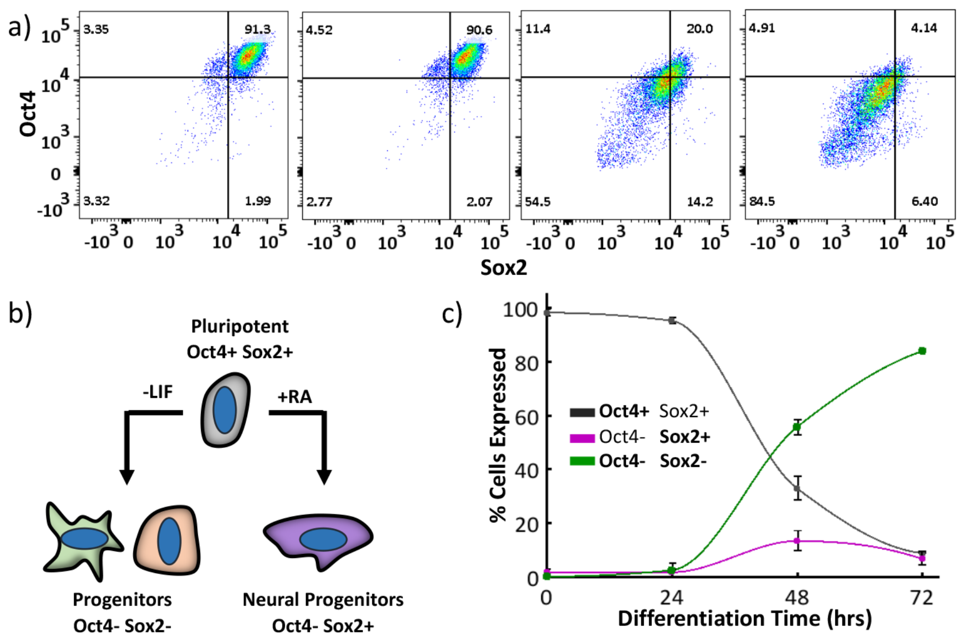

Supplementary Figure 1: Sox2 and Oct4 concomitant loss through LIF withdrawal in the presence of serum containing media (a) characterized by flow cytometry. (b) Schematic representing the change in gene expression towards the predominant progenitor state in ESC populations during two differentiation protocols, LIF-withdrawal in serum containing media and retinoic acid addition. (c) Quantification of flow cytometry data in (a), with a large population of Oct4-Sox2- progenitors accumulating between 24 and 72 hours in addition to a smaller population of Oct4-Sox2+ cells, n=9 (3 biological replicates, 3 technical replicates for each biological replicate).

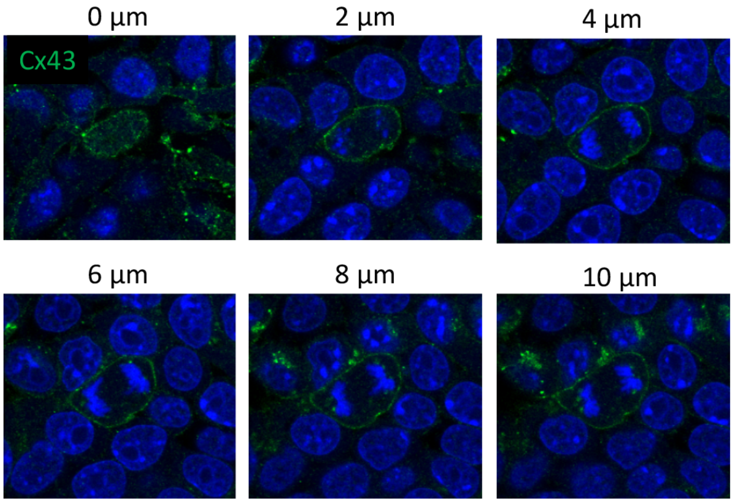

Supplementary Figure 2: Consistent Cx43 stain throughout membrane in mitotic cells. A confocal z-stack shows a diffuse ‘ring’ structure at each focal plane in the membrane of mitotic cells while non-mitotic cells display a more punctate staining profile.

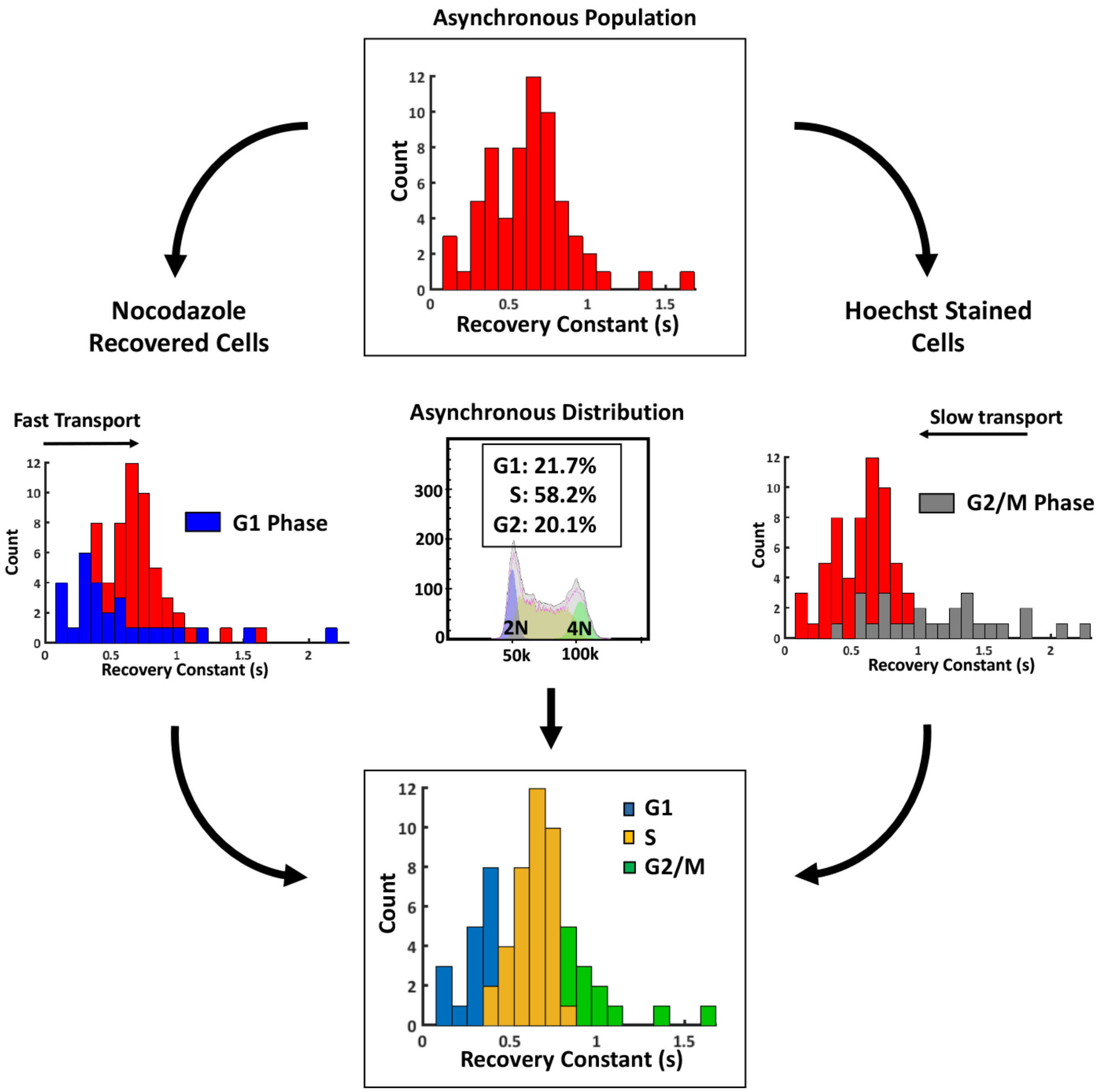

Supplementary Figure 3: Defining the asynchronous distribution of recovery constants by cell cycle state. Through identification of mitotic cells using Hoechst-staining, it was discovered that the recovery constants of G2/M phase cells cluster along the right tail of the asynchronous distribution. Similarly, performing GAP-FRAP on a nocodazole-recovered (NOC45) population yielded a cluster along the left tail of the asynchronous population and a few cells with slower transport. Since the NOC45 population consists predominantly of G1- and G2/M-phase cells (see Fig 3a) and G2/M-phase were shown to have decreased transport rates, we can associate the cluster along the left tail with G1-cells. Using the distribution of cells in each cell cycle state within an asynchronous population, the recovery constants can be divided by: 21.7% of the fastest transporting cells were in G1-phase, 20.1% of the slowest transporting cells were in G2/M-phase, and the remaining 58.2% were in S-phase.

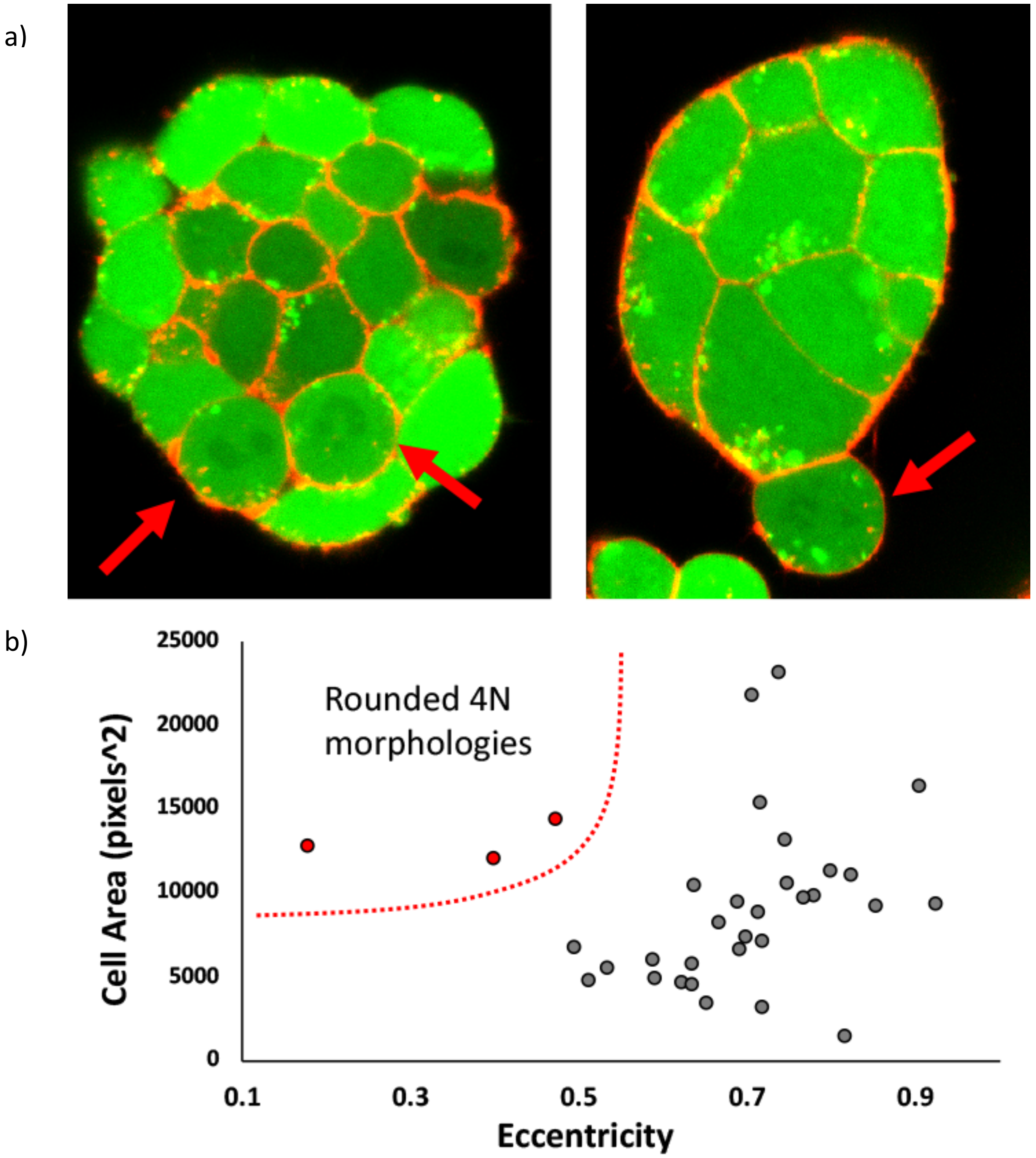

Supplementary Figure 4: Rounded morphology of mitotic cells in the nocodazole recovered (NOC45) population that coincides with slower transport (a). The morphological features of mitotic cells can be described by eccentricity and cell area, with mitotic cells diverging from the population in (a, illustrated by red arrows) with low eccentricity and above average cell area.

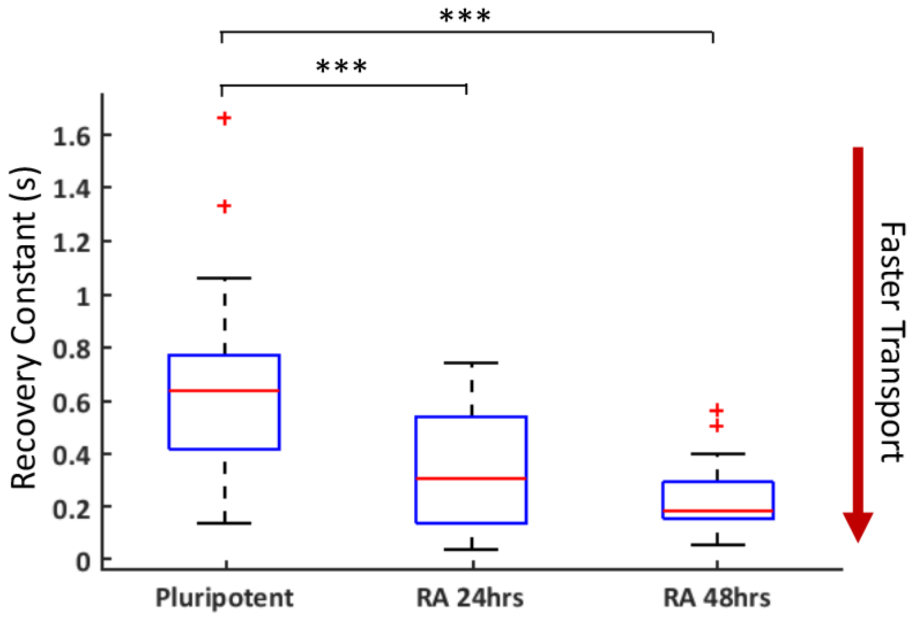

Supplementary Figure 5: Comparison of recovery constants collected over the first 48 hours of retinoic acid (RA) exposure that were calculated from GAP-FRAP results. The measured cells were randomly selected within the populations at each time point. At both 24 and 48 hours of RA-treatment, there was a statistically significant ($p < 1e-5$) increase in the rate of intercellular transport compared to the pluripotent population, denoted by the decrease in the recovery constant. By 48 hours, the recovery constants showed less overall variance in comparison to the pluripotent and 24 hour RA populations. Box plots show the median, the 25th and 75th percentiles, Tukey whiskers (median ± 1.5 times interquartile range), and outliers (+).

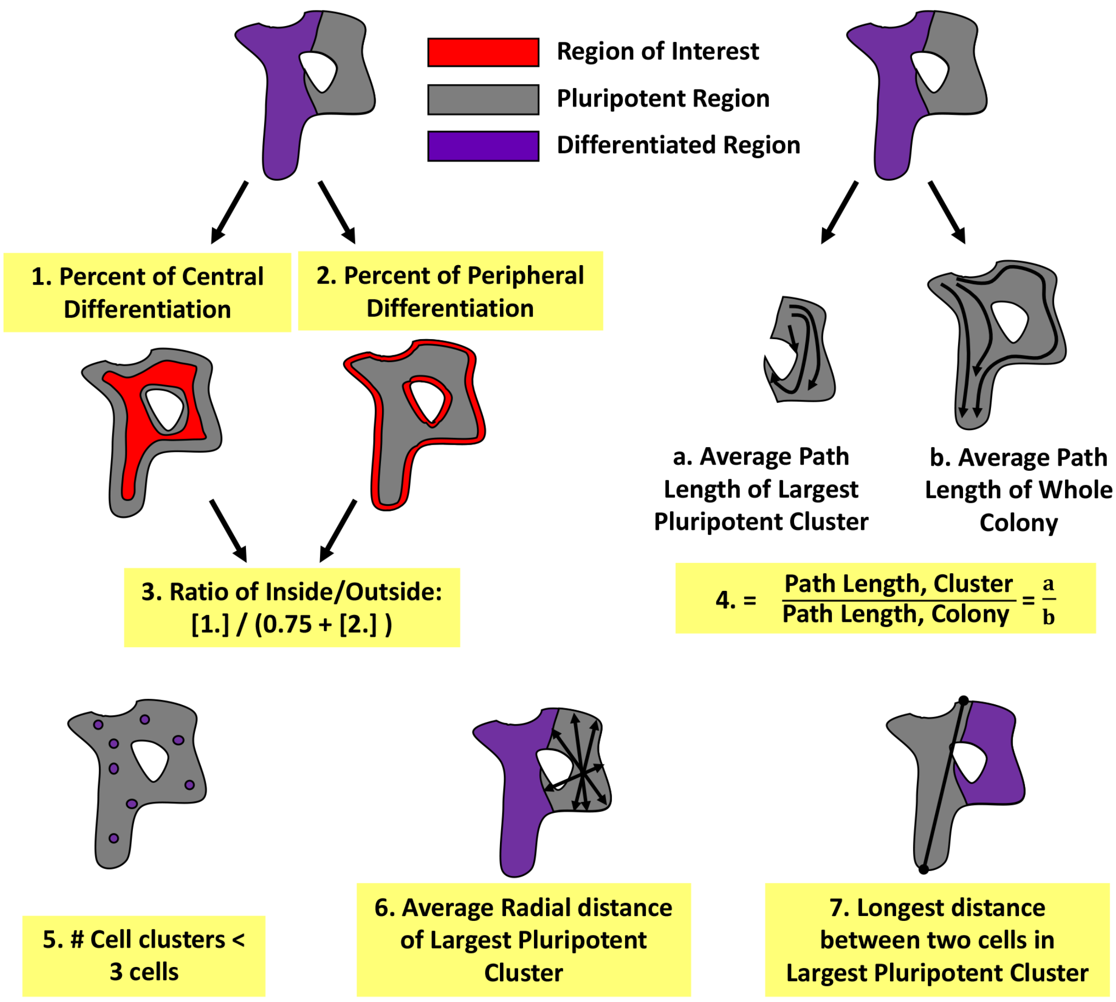

Supplementary Figure 6: The seven selected metrics used to quantify the spatial patterning within colonies. Each metric is represented by a single numerical value and every sample (experimental & computational) is depicted by the same seven metrics. Colony-based metrics: (1,2,3,5), Cluster-based metrics: (4,6,7).

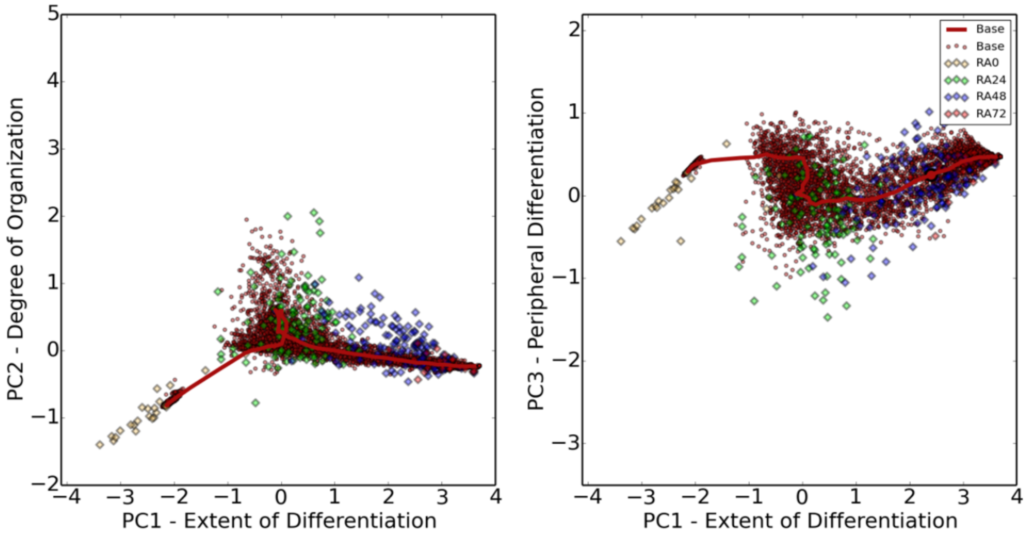

Supplementary Figure 7: Experimental trajectory data points overlaid on data cloud of every simulation data point used to compute average trajectory. The simulation data cloud encompasses the regions containing the highest density of experimental data points and has a similar range of values along each principle component.

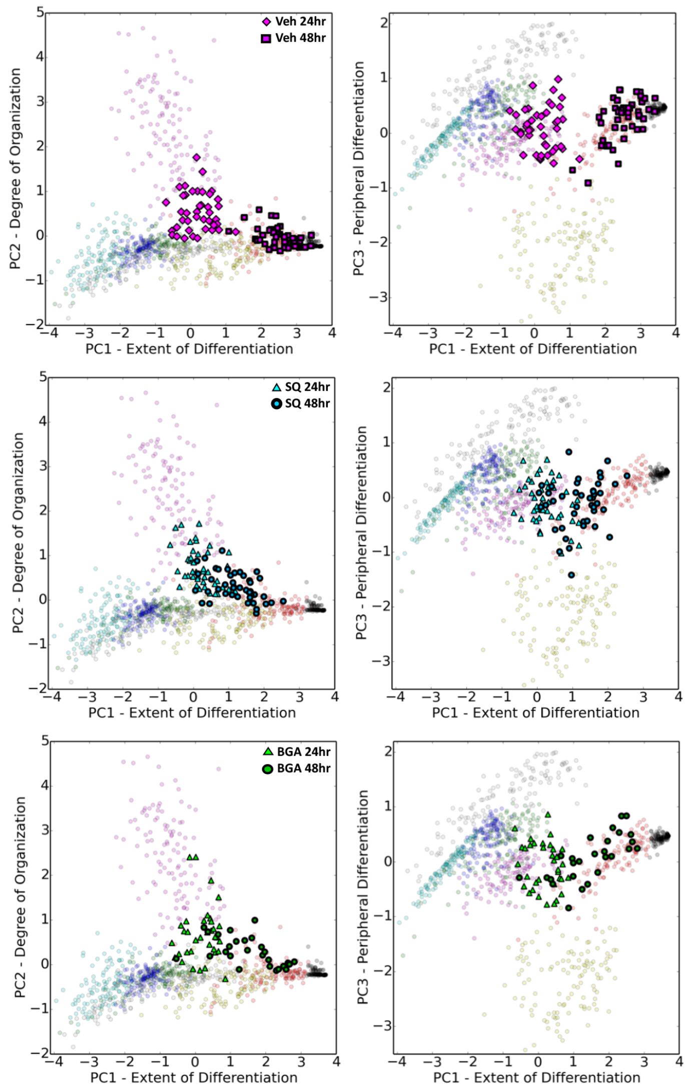

Supplementary Figure 8: D3 experimental colonies at 24 and 48 hour time points for the Vehicle control (n=87), SQ-treatment (n=87), and BGA-treatments (n=62) converted into latent space. Averages of these values are illustrated in Figure 6.

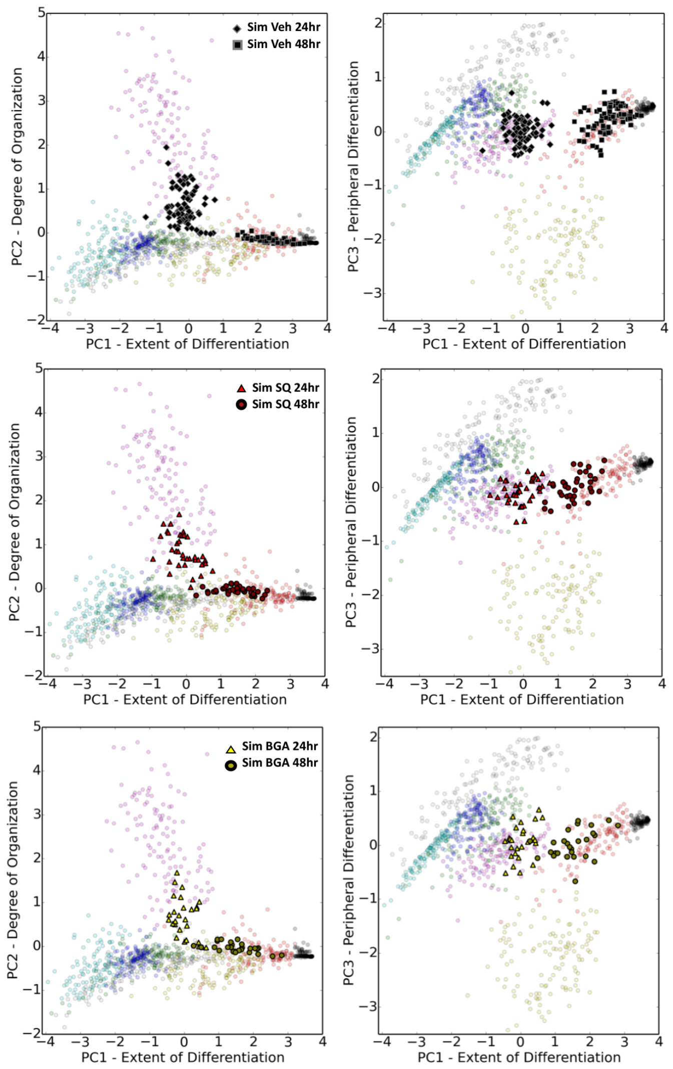

Supplementary Figure 9: Simulation colonies at 24 and 48 hour time points for the Vehicle control (n=75), SQ-treatment (n=30), and BGA-treatments (n=30 converted into latent space. Averages of these values are illustrated in Figure 6.

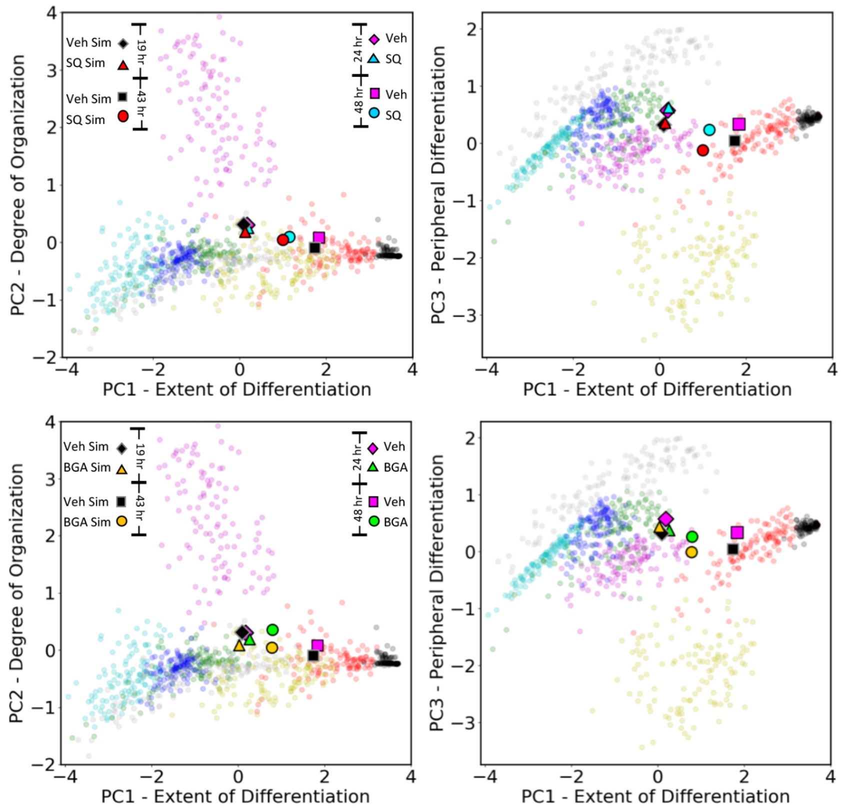

Supplementary Figure 10: Perturbation to the intercellular network of a multicellular RW4 ESC population affects neural differentiation in a temporal manner. Similarly to Fig 6, neither adenylyl cyclase (AC) inhibition (a, SQ-treatment) nor gap junction (GJ) inhibition (b, BGA-treatment) induced a significant change in spatial or temporal characteristics of differentiation compared to the vehicle control at 24 hours. By 48 hours, a temporal shift along PC1 was observed for both treatments (a,b), but with a more significant decrease in the differentiation rate for GJ-inhibition. Furthermore, GJ-treatment at 48 hours produced an increase in stochasticity compared to the vehicle and SQ-treatment groups. In comparison to Fig 6, the RW4 tended to differentiate in a more "inside-out" manner than the noted 'outside-in' differentiation of the D3 ESC line. Interestingly, the intercellular model suggests that RW4 cells differentiate slower than D3 cells in response to RA, as indicated by the accurate prediction of both treatments at the simulated 19 and 43 hour time points. (Veh: n=59, SQ: n=49, BGA: n=46)

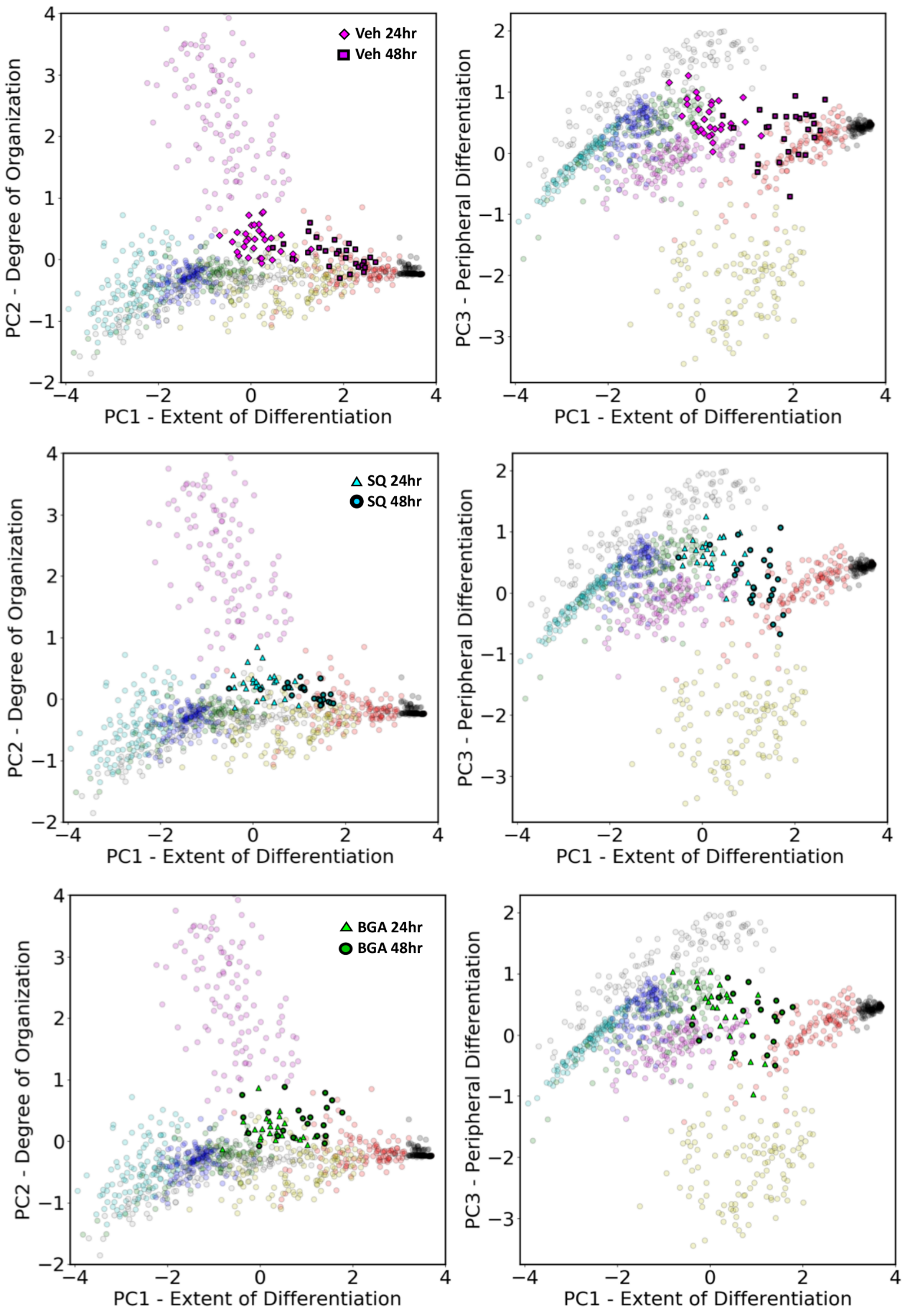

Supplementary Figure 11: RW4 experimental colonies at 24 and 48 hour time points for the Vehicle control (n=59), SQ-treatment (n=49), and BGA-treatments (n=46) converted into latent space. Averages of these values are illustrated in Figure S10.

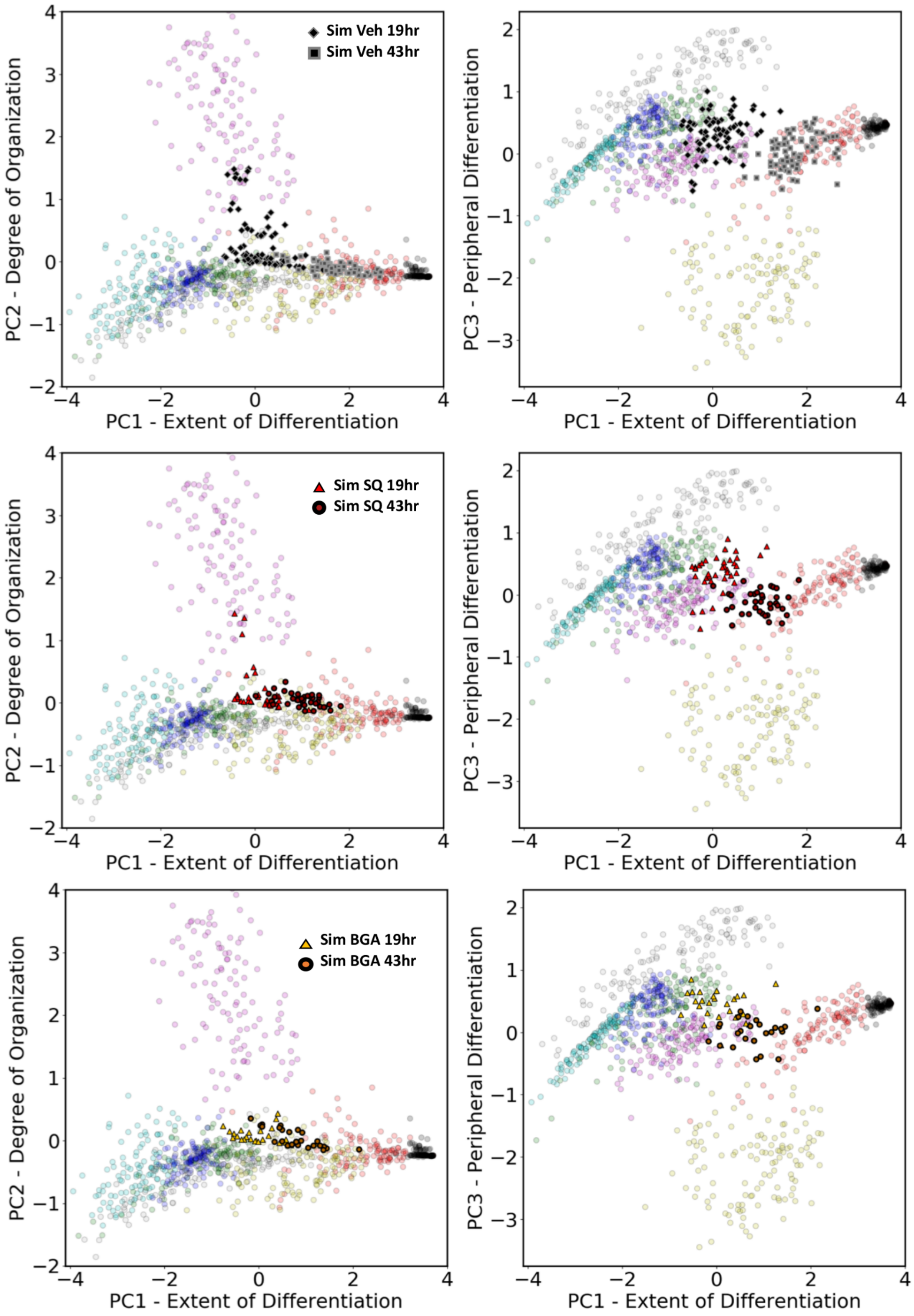

Supplementary Figure 12: Simulation colonies at 19 and 43 hour time points for the Vehicle control (n=75), SQ-treatment (n=30), and BGA-treatments (n=30) converted into latent space. Averages of these values are illustrated in Figure S10.

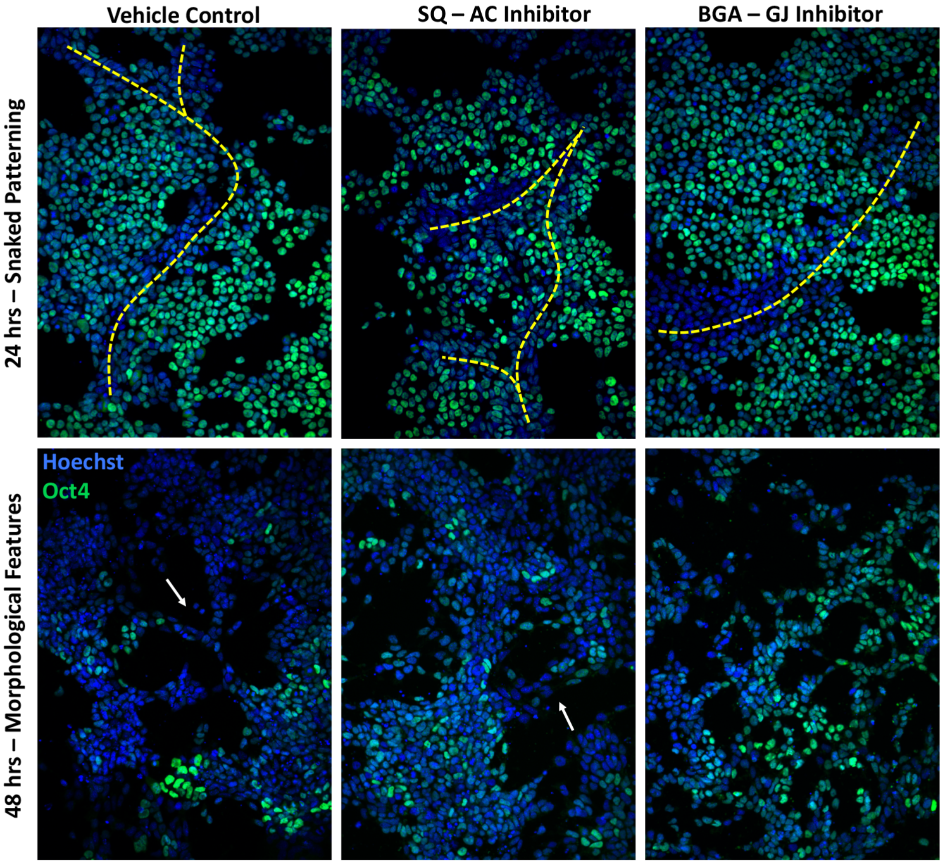

Supplementary Figure 13: RW4 colony patterning and morphology after 24 and 48 hours of retinoic acid exposure, respectively, with adenylyl cyclase or gap junction inhibition compared to the vehicle control. (a) At 24 hours, obvious examples of the snaked pattern class emerge (demonstrated by yellow dotted lines). (b) At 48 hours, both the Vehicle and SQ colonies tend to form larger cell clusters with a few smaller extensions between clusters (indicated by white arrows). In contrast, after GJ inhibition the majority of cells in the population form a network of branching extensions and develop numerous circular pockets.

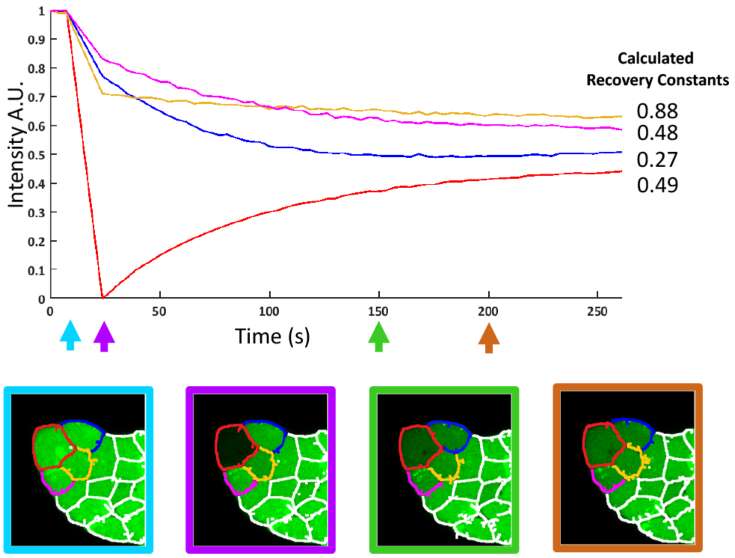

Supplementary Figure 14: Quantifying the loss of fluorescence in the cells adjacent to the photobleached cell during gap-FRAP. In the circumstance where the change in intensity of primary neighbors is sufficient to measure, the relative transport of those cells can be quantified in a similar manner to typical FRAP experiments. Interpretation of these transport rates is colony specific because non-photobleached cells are experiencing both a loss of fluorescence into the photobleached cell and a gain of fluorescence from other adjacent cells.

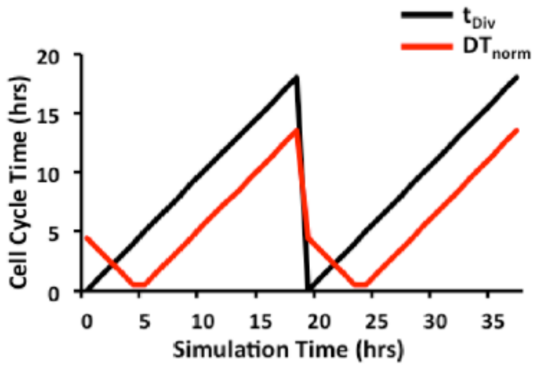

Supplementary Figure 15: Comparison of cell cycle time and the respective DTnorm transform.

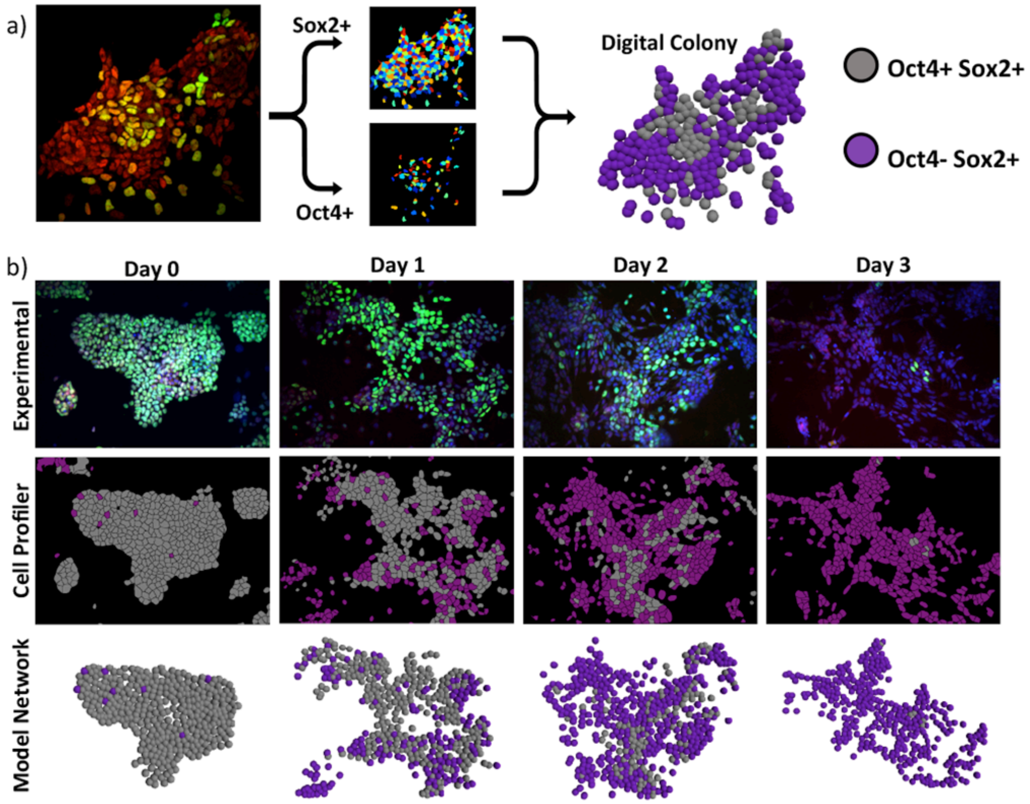

Supplementary Figure 16: CellProfiler pipeline used to convert experimentally imaged colonies to digital networks synonymous with model outputs. (a) Conversion of a single image using Sox2 and Oct4 staining into a digital colony. (b) Experimental images, with digitally enhanced Oct4 stain for ease of comparison, before conversion to CellProfiler representations and respective digital networks.
