# Supplementary material for: Dynamic intercellular transport modulates the spatial patterning of differentiation during early neural commitment
Source: Nat Commun. 2018 Oct 5;9:4111. doi: 10.1038/s41467-018-06693-1 (PMC6173785; doi:10.1038/s41467-018-06693-1)
Supplement: Supplementary file 2 — Description of Additional Supplementary Files [file 41467_2018_6693_MOESM2_ESM.pdf]

Description of Additional Supplementary Files

File Name: Supplementary Movie 1
Description: Asynchronous cell cycle implemented in colony growth and differentiation

File Name: Supplementary Movie 2
Description: Synchronized cell cycle implemented in colony growth and differentiation
